# Oxidative Stress Induced Senescent Macrophage‐Driven Squamous Cell Carcinoma Invasion via Glutamine Metabolic Reprogramming

**DOI:** 10.1111/acel.70592

**Published:** 2026-06-23

**Authors:** Shimeng Wang, Jingtian Mu, Wei Zhao, Can Hu, Xueke Shi, Hongmei Zhou, Junjiang Liu, Fanglong Wu

**Affiliations:** ^1^ State Key Laboratory of Oral Diseases & National Center for Stomatology & National Clinical Research Center for Oral Diseases & Frontier Innovation Center for Dental Medicine Plus, Department of Oral Medicine, West China Hospital of Stomatology Sichuan University Chengdu Sichuan China; ^2^ Department of Pathology and Laboratory Medicine, School of Medicine University of California, Davis Sacramento California USA; ^3^ Department of Stomatology Affiliated Hospital of North Sichuan Medical College Nanchong China

**Keywords:** glutamine metabolism reprogramming, oxidative stress, senescence‐associated secretory phenotype, senescent macrophages, squamous cell carcinoma

## Abstract

Oxidative stress drives tumor microenvironment (TME) remodeling by inducing metabolic reprogramming and cellular senescence. Glutamine, a key substrate supporting oxidative stress defense, has been implicated in TME remodeling and metastasis, yet its specific role in initiating tumor invasion remains unclear. Here, oxidative stress induced the generation of senescent macrophages in the TME, and clinical samples showed that their accumulation positively correlates with malignancy. We established cisplatin‐ and radiation‐induced senescent macrophage models that exhibited distinct senescence‐associated secretory phenotypes (SASP) and enhanced squamous cell carcinoma (SCC) migration and invasion. Integrated metabolomic and transcriptomic analyses revealed the glutamine–glutamate pathway as a central metabolic hub, with glutaminase 2 upregulated to drive glutaminolysis and strongly associated with IL‐1β expression. Mechanistically, IL‐1β secreted by senescent macrophages promoted tumor invasion by downregulating IL‐1R2 and activating NF‐κB signaling in SCC cells. Targeting the glutamine metabolism–regulated IL‐1β/IL‐1R2 axis effectively suppressed SCC invasion. These findings uncover a novel metabolic mechanism linking glutamine metabolism to SASP regulation and suggest a therapeutic strategy to limit SCC invasion.

## Graphical abstract

Oxidative stress induces GLS2‐dependent IL‐1β secretion in senescent macrophages, promoting SCC invasion through the IL‐1β/IL‐1R2 axis. Targeting IL‐1β suppresses invasion more effectively than GLS2 inhibition, highlighting a potential therapeutic strategy for SCC.

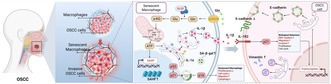

**Abbreviations**
53BP1: p53‐bindingp53 binding protein 1
ADSSL1: adenylosuccinate synthase like 1
CM: conditionedconditional medium
EMT: epithelial‐mesenchymal transition
GLS2: glutaminase 2
GM‐CSF: granulocyte‐macrophage colony‐stimulating factor
GSEA: gene set enrichment analysis
H3K9me: histone H3 lysine‐9 methylation
IHC: immunohistochemical
IL‐1R: IL‐1 receptor
KEGG: Kyoto Encyclopedia of Genes and Genomes
LPS: lipopolysaccharide
MDSCs: myeloid‐derived suppressor cells
mIHC: immunohistochemical
OLK: oral leukoplakia
OSCC: oral squamous cell carcinoma
pH2AX: histone family 2A variant X
RNA‐seq: RNA sequencing
ROS: (reactivereactive oxygen species)
SASP: senescence‐associated secretory phenotype
SA‐β‐gal: senescence‐associated β‐galactosidase
scRNA‐seq: single‐cell RNA sequencing
SEM: scanning electron microscopy
TEM: transmission electron microscopy
TME: tumortumour microenvironment
Tregs: T regulatory cells

## 1 Introduction

Squamous cell carcinoma (SCC) is a highly invasive malignancy arising from squamous epithelium and is characterized by substantial morbidity and metastasis (Li et al. 2019; Yan et al. 2011). Metastasis remains the leading cause of cancer‐related mortality and signifies the terminal phase of tumor progression. Because local invasion is the essential first step enabling metastatic dissemination (Graham and Shibata 2020), clarifying the early events of SCC metastasis is critical for the development of effective preventive strategies.

Metabolic reprogramming is a defining hallmark of tumors. Among metabolic pathways, (Hanahan 2022) glutamine metabolism has attracted considerable attention because of its dual function as a carbon and nitrogen source, estalishing its role as a “carbo–nitrogen hub” (Cluntun et al. 2017). Through glutaminolysis, glutamine supplies α‐ketoglutarate to the tricarboxylic acid (TCA) cycle to support bioenergetics and anabolic processes, while glutathione (GSH) and nicotinamide adenine dinucleotide phosphate (NADPH) production to counter oxidative stress and preserve redox homeostasis (Gong et al. 2022; Jin et al. 2016). Tumors frequently exhibit pronounced glutamine dependence, or “glutamine addiction” (Quek et al. 2022; Zou et al. 2025). Aberrant reprogramming of glutamine metabolism not only sustains cancer cell proliferation and survival, but also drives tumor microenvironment (TME) remodeling, including activating cancer‐associated fibroblasts, enhancing immunosuppressive myeloid‐derived suppressor cell and Tregs functions, and promoting tumor‐supportive macrophage polarization (Zou et al. 2025; Ma et al. 2022; Hu et al. 2023). Consequently, glutamine metabolism maintains cellular homeostasis while shaping a microenvironment that fosters tumor migration and invasion, positioning it as a major metabolic driver of the metastatic cascade.

Oxidative stress is another central force in TME remodeling. It forms a positive feedback loop with glutamine metabolism to enhance tumor adaptability and influences the fate of multiple cell types through reactive oxygen species (ROS) accumulation. Tumor‐infiltrating macrophages, in particular, exhibit notable heterogeneity in origin, differentiation, and secretory profiles (Kloosterman and Akkari 2023; Liebold et al. 2024). Their secretomes guide organotropic metastasis by facilitating pre‐metastatic niche formation through exosomes, chemokines, and the release of other modulatory factors. Under distinct stimuli, mature macrophages typically polarize toward M1 (classically) or M2 (alternatively) phenotypes, contributing to tumor suppression in early stages or tumor promotion in late/advanced stages, respectively (Kloosterman and Akkari 2023; Duan and Luo 2021). We previously demonstrated that macrophages enhance cancer expansion, invasion, and stemness through direct interactions with tumor cells (Gomez et al. 2020; Wu et al. 2019). Senescent macrophages in particular have been implicated in tumor initiation (Walters 2023), although their effects on cancer invasion remain unknown. Evidence indicates that excessive ROS can drive macrophages toward senescence (Wang et al. 2019; Danish et al. 2025), suggesting oxidative stress as an important inducer of macrophage senescence and a potential determinant of early invasive behavior.

During senescence, macrophages undergo genome‐wide alterations, canonical biomarker activation, and assembly of a paracrine‐competent senescence‐associated secretory phenotype (SASP) (Muñoz‐Espín and Serrano 2014). Increasing evidence highlights SASP as a key mediator linking senescent cells to TME remodeling and tumor progression (D'Ambrosio and Gil 2023; Dong et al. 2024). Notably, recent studies have demonstrated that senescent macrophages accumulate in premalignant lesions and contribute to malignant transformation via SASP‐mediated signaling in lung carcinogenesis (Prieto et al. 2023; Haston et al. 2023; Zheng et al. 2025). These observations suggest that macrophage senescence is not merely a bystander effect but an active contributor to tumorigenesis. However, the mechanisms through which glutamine metabolism regulates SASP remain insufficiently defined. Our study demonstrates that oxidative stress–induced senescent macrophages undergo glutamine metabolic reprogramming and promote SCC invasion through SASP enhancement. Inhibiting glutamine‐dependent IL‐1β hypersecretion reduced invasion, revealing the glutamine–SASP axis as both a mechanistic insight and a promising metabolic target for anti‐invasion therapies.

## 2 Results

### 2.1 Increased Infiltration of Senescent Macrophages Correlates With Malignancy in Human SCC

**FIGURE 1:**
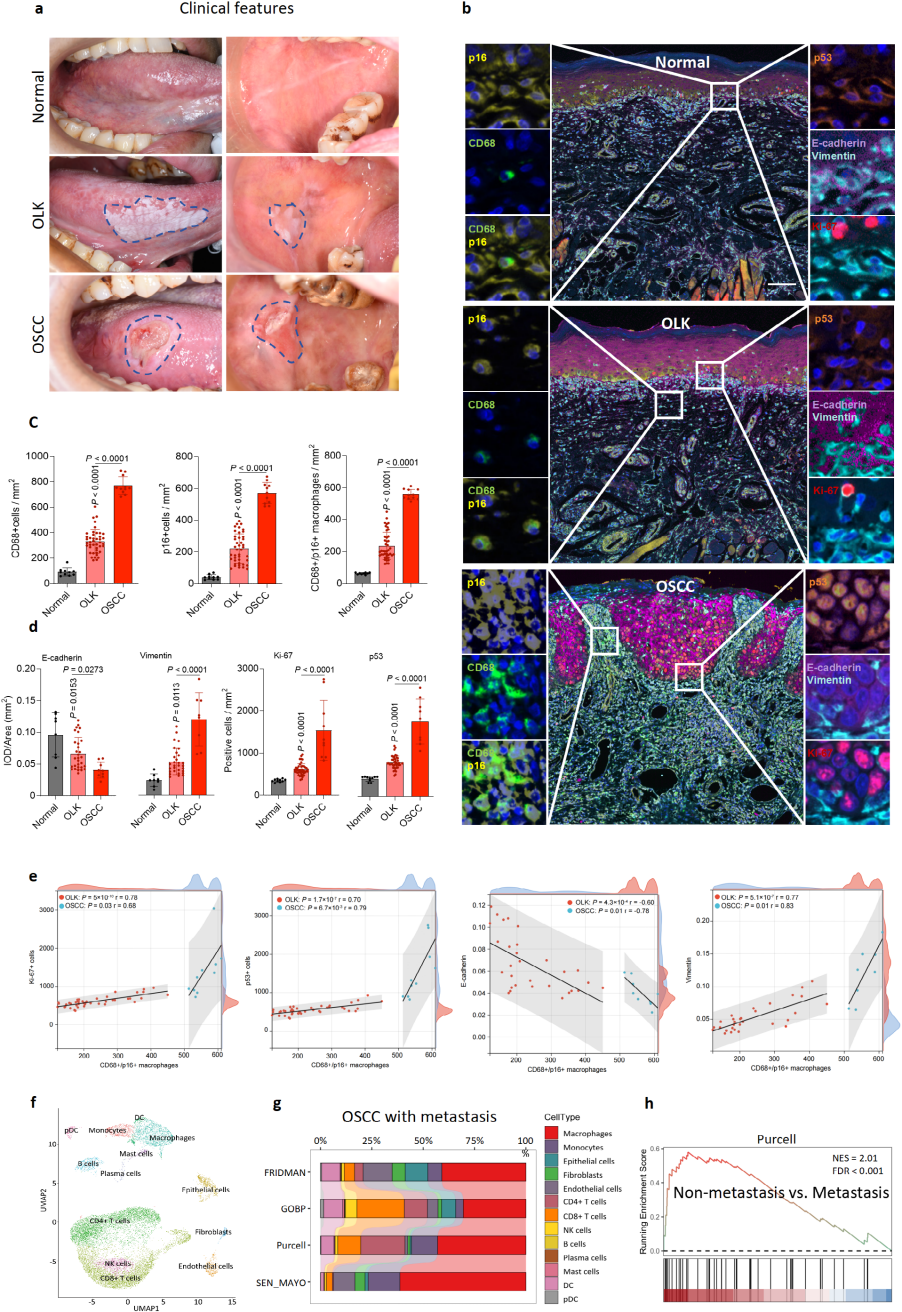
Senescent macrophages are closely related to human oral squamous cell carcinoma progression. (a) Clinical images are representative of human normal oral mucosa, oral leukoplakia (OLK) and oral squamous cell carcinoma (OSCC) in the tongue and buccal areas. (b) Representative 7‐color multiplex immunofluorescence (mIF) images showing the co‐expression of CD68, p16, p53, Ki‐67, E‐cadherin and Vimentin proteins in human oral normal mucosa, OLK, and OSCC patients. Scale bars, 100 μm. (c) Quantification of CD68, p16, and CD68/p16 in different human oral samples from each patient (CD68/p16 positive cells: *N* = 10 in normal, *n* = 43 in OLK, *n* = 11 in OSCC; CD68 and p16 positive cells: *N* = 9 in normal, *n* = 43 in OLK, *n* = 11 in OSCC). (d) Quantification of Ki‐67, p53, E‐cadherin and Vimentin in different human oral samples from each patient (Ki‐67/p53/E‐cadherin/Vimentin positive cells: *N* = 10/10/9/9 in normal, *n* = 42/42/30/30 in OLK, *n* = 9/9/9/8 in OSCC). (e) Pearson correlation analysis was performed to analyze the relationships between CD68+/p16+ senescent macrophages and Ki‐67+, p53+, Vimentin+, and E‐cadherin+ cells. (f) Uniform manifold approximation and projection (UMAP) of single‐cell RNA sequencing (scRNA‐seq) of all cells from metastasis OSCC, identifying 13 clusters. (g) Gene Set Enrichment Analysis (GSEA) results showing that these cells in scRNA‐seq data were annotated using gene module scores for senescent cells in the FRIDMAN Senescence, GOBP Cellular Senescence, Purcell and Sen MAYO datasets. (h) GSEA enrichment plot of bulk RNA sequencing of senescence‐associated genes between non‐metastasis OSCC and OSCC with metastasis in Purcell sets. Statistical significance was determined by permutation analysis. The percentage of positive cells was determined by one‐way ANOVA with Tukey's post hoc test (c, d).

To clarify the role of senescent macrophages in SCC, we used co‐expression of CD68^+^/p16^INK4a+^/p53/E‐cadherin/Vimentin/Ki67 as the defining marker. Representative 7‐color multiplex immunofluorescence (mIF) images showed that, compared with normal and precancerous tissues, OSCC stroma exhibited extensive infiltration of senescent cells, with senescent macrophages being the most prominent population. In OSCC, the expression of Ki‐67 and p53 was markedly elevated within the epithelium, and the epithelial–stromal boundary appears blurred (Figure 1a,b). The same pattern was independently validated in the CD68/p16, CD68/p16/Ki‐67, and CD68/p16/p53 multiplex co‐staining assays (Figure S1b–d). Quantitative analysis showed a stepwise and statistically significant increase in CD68^+^/p16^INK4a+^ senescent macrophage infiltration across human oral normal mucosa, oral leukoplakia (OLK), and oral SCC (OSCC) (Figure 1c). Because epithelial‐mesenchymal transition (EMT) is central to tumor invasion and metastasis (Glaviano et al. 2025), we assessed EMT‐related proteins, including Vimentin and E‐cadherin. Quantitative immunohistochemistry (IHC) demonstrated increased Vimentin expression and decreased E‐cadherin expression during SCC progression (Figure 1d; Figure S1a). Together, these findings indicate that senescent macrophage accumulation may be a strong predictor of SCC malignancy.

Given the established roles of senescent stroma in shaping cancer cell behavior (Assouline et al. 2024; Mori et al. 2024), we hypothesized that senescent macrophages similarly influence SCC progression. Pearson correlation analysis showed that Vimentin, Ki‐67, and p53 were positively associated with CD68+/p16^INK4a+^ senescent macrophages, whereas E‐cadherin was negatively associated with CD68+/p16^INK4a+^ senescent macrophages in OLK and OSCC tissues (Figure 1e). To support these observations, we performed single‐cell RNA sequencing (scRNA‐seq) and identified 17 cell clusters representing 13 annotated cell types in human OSCC tissues (Figure 1f; Figure S1e,f). Immune cells were more abundant than epithelial cells or stromal cells. Single‐cell compositional bar plots revealed a marked dominance of senescent macrophages across tumor samples and a corresponding suppression of anti‐tumor immunity, including reduced infiltration of senescent CD8+ T cells (Figure 1g). Moreover, senescence‐associated gene sets were significantly upregulated in OSCC with metastasis compared with non‐metastatic tumors (Figure 1h; Figure S1g). Collectively, these findings support a promotive role of senescent macrophages in OSCC progression, particularly in regulating OSCC invasion and metastasis.

### 2.2 Oxidative Stress Leads to Macrophage Senescence and Adaptive Changes

**FIGURE 2:**
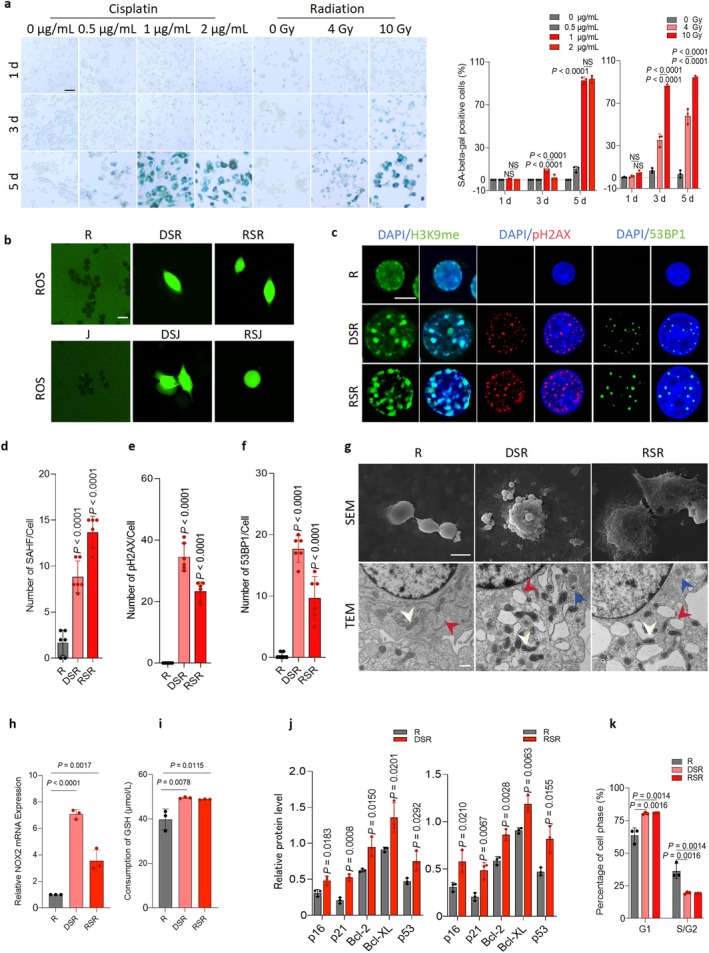
Oxidative stress damage caused by cisplatin or radiation induces senescence and adaptive changes in macrophages. (a) RAW264.7 cells (R) were treated with cisplatin (drug‐induced senescent RAW264.7 cells, DSR) or radiation (radiation‐induced senescent RAW264.7 cells, RSR) and stained for SA‐β‐Gal. Quantitation of the percentage of SA‐β‐Gal+ cells. Scale bars, 100 μm. (b) Representative immunofluorescence images for reactive oxygen species (ROS) in the senescent macrophages and controls (top: R, DSR, and RSR, bottom: J, DSJ, and RSJ). Scale bar, 20 μm. (c‐f) Representative fluorescence images of H3K9me, pH2AX and 53BP1. Quantitation of H3K9me (d), pH2AX (e) and 53BP1 (f). Scale bars, 10 μm. (g) Top: Scanning electron microscopy (SEM) images showing more phagocytic vacuoles than the controls. Scale bar, 200 μm. Bottom: Transmission electron microscopy (TEM) images showing mitochondrial shrinkage with increased electron density (white arrow), endoplasmic reticulum expansion with degranulation (red arrow) and phagocytic vacuoles (blue arrow). Scale bar, 500 nm. (h) Relative mRNA expression of NOX2 in DSR and RSR from RAW264.7 cells, respectively. (i) Concentration of GSH in R, DSR or RSR, respectively. (j) Relative protein expression of senescence‐associated proteins, including p16, p21, p53, Bcl‐2, and Bcl‐XL. (k) Histogram of flow cytometry with PI staining to address the cell cycle of senescent macrophages showing the cell proportions in different phases of G1 and G2/S. *n = 3* each group from independent biological replicates (a, h, i, j, k), *n = 6* each group for independent biological replicates (d, e, f). Statistical significance was determined by one‐way ANOVA with Tukey's post hoc test and the data are presented as the means ± s.d. (a, d, e, f, h, i, j, k).

Next, we established three in vitro oxidative stress–induced macrophage senescence models to investigate the role of macrophages in SCC invasion. Several agents, including D‐galactose, radiation, lipopolysaccharide, tamoxifen, acrylamide, and *Pseudomonas aeruginosa* , have been reported to induce oxidative stress (Petrova et al. 2016; Herranz and Gil 2018). Accordingly, we triggered senescence in RAW264.7 and J774A.1 macrophages using D‐galactose, cisplatin, or ionizing radiation (Figure S2a). Because pH 6.0 β‐galactosidase (SA‐β‐gal) is a canonical senescence biomarker (Lee et al. 2006), we assessed SA‐β‐gal activity in cisplatin‐ and radiation‐induced senescent macrophages and found that a 5‐day exposure to 1 μg/mL cisplatin or 10 Gy radiation most effectively induced SA‐β‐gal^+^ macrophages in a dose‐ and time‐dependent manner (Figure 2a; Figure S2b,d). Exposure of RAW264.7 macrophages to 10 mg/mL D‐galactose also induced SA‐β‐gal^+^ cells (Figure S3a). Quantitative analysis of western blotting (WB) showed upregulation of p16, p21, Bcl‐2, Bcl‐XL, and p53 in senescent macrophages, with the exception of p53 in cisplatin‐treated J774A.1 cells and p16 in D‐galactose–treated RAW264.7 macrophages (Figure 2j; Figure S3c–e). These findings suggest that cisplatin, radiation, and D‐galactose serve as robust, dose‐dependent triggers of macrophage senescence.

We then assessed ROS accumulation and associated senescence damage, including DNA damage, and mitochondrial dysfunction, ROS levels were markedly elevated in senescent macrophages (Figure 2b). NADPH oxidase 2 (NOX2), a key enzyme for ROS generation, showed significantly increased expression following cisplatin or radiation exposure (Figure 2h). Nuclear DNA damage markers—histone H3 lysine‐9 methylation (H3K9me), phosphorylation of the histone family 2A variant X (pH2AX), and p53‐binding protein 1 (53BP1)—were also significantly elevated (Figure 2c–f; Figures S2c,e and S3b). Transmission electron microscopy revealed mitochondrial fragmentation, reduced organelle size, increased membrane density, and outer membrane discontinuities, consistent with oxidative injury (Figure 2g; Figure S2g). Notably, intracellular glutathione (GSH) also increased significantly in senescent macrophages (Figure 2i). Collectively, these results show that cisplatin and radiation induce oxidative stress, initiating macrophage senescence and adaptive metabolic responses.

Further, senescent macrophages exhibited cellular hypertrophy ultrastructural senescence features under inverted microscopy and scanning electron microscopy, including surface disorganization and abundant phagocytic vacuoles (Figure 2g; Figure S2g,l,m). To assess phagocytosis, we confirmed that cisplatin‐ and radiation‐induced senescence significantly augmented phagocytic capacity in both RAW264.7 and J774A.1 macrophages (Figure S2h–i). Furthermore, we explored cell cycle progression, proliferation, and migration. Flow cytometry revealed a consistent shift toward G1‐phase accumulation with G2/S depletion across all senescent models (Figure 2k; Figures S2f and S3h). CCK‐8 assays demonstrated that 1 μg/mL cisplatin, 10 Gy radiation, and 10 mg/mL D‐galactose induced macrophage senescence while suppressing proliferation (Figure S3i–k). Additionally, cisplatin and radiation enhanced macrophage migratory capacity (Figure S2j–k). Taken together, these findings indicate that D‐galactose‐, cisplatin‐, or radiation‐induced senescence produces a coordinated phenotype characterized by structural derangements and functional alterations. Furthermore, to determine whether the senescent macrophages exhibit M1 or M2 characteristics, we assessed the expression of representative markers (CD86, TNF‐α and iNOS for M1, CD206, IL‐10, and arginase 1 for M2). The results showed reduced expression of CD86 and IL‐10, accompanied by increased expression of iNOS and arginase 1 (Figure S3l).

### 2.3 Glutamine Metabolic Reprogramming of Senescent Macrophages Regulated by Glutaminase 2 (GLS2)

**FIGURE 3:**
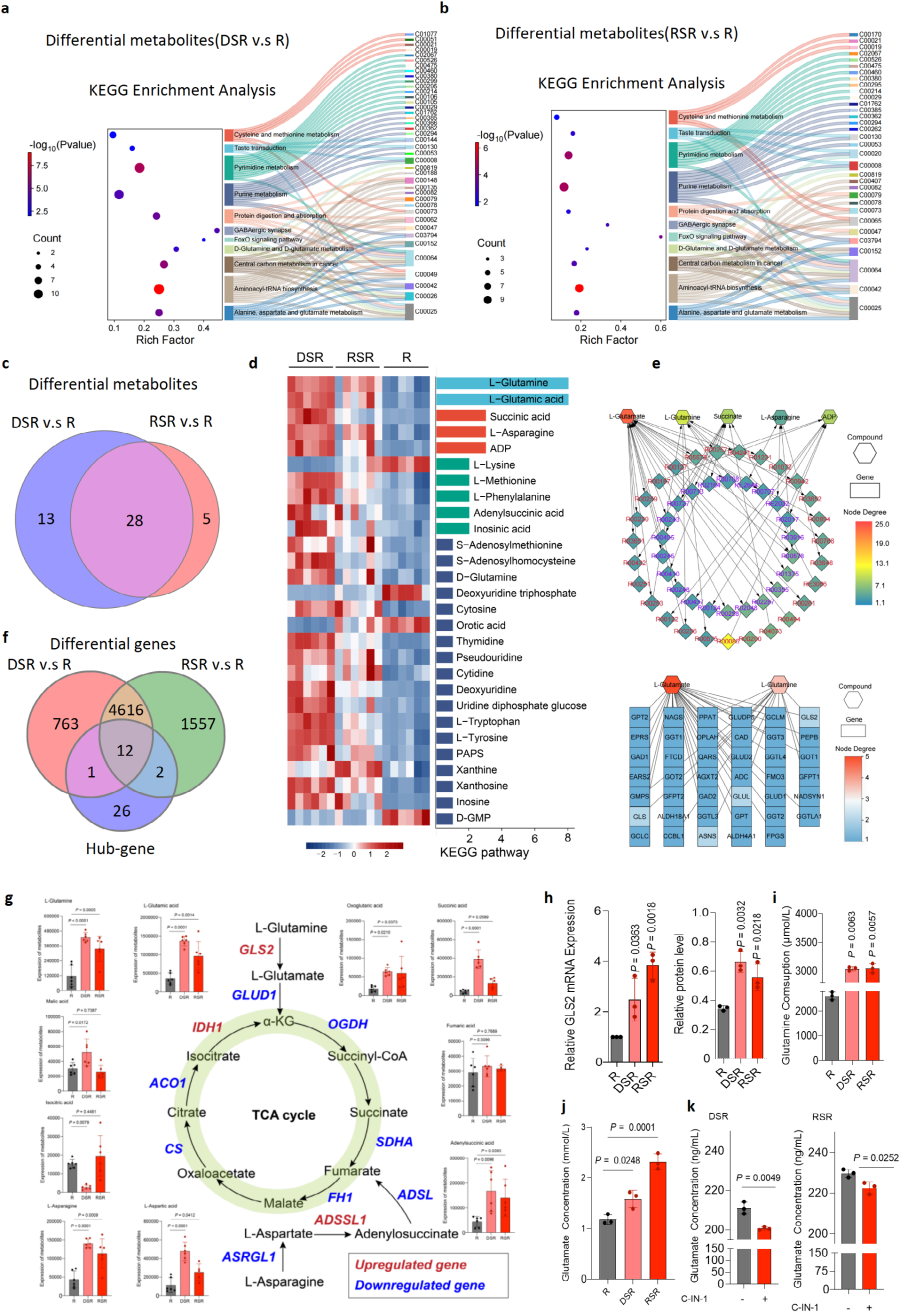
Glutamine metabolism in senescent macrophages is enhanced and regulated by GLS2. (a, b) Sankey diagrams illustrating metabolic alterations in senescent macrophages. In the DSR group, 37 altered metabolites were mapped to 11 metabolic pathways, while in the RSR group, 30 altered metabolites were associated with 11 pathways. Colors and labels highlight the close link between L‐glutamine and L‐glutamate (left) and glutamine metabolism (right). (c) Venn diagram showing the overlap of significantly altered metabolites associated with central carbon metabolism, alanine metabolism, aspartate metabolism, and glutamine metabolism in DSR or RSR. (d) Heat map of the top 28 altered metabolites from DSR or RSR (*n* = 6). (e) Bubble plots showing the results of gene‐metabolite KEGG joint enrichment analysis comparing the differential metabolites in senescent macrophages (DSR and RSR) with the control (R). The color of the points represented false discovery rate (FDR). (f) Venn diagram showing overlap of the significantly altered GLS2 gene related to central carbon metabolism, alanine metabolism, aspartate metabolism, and glutamine metabolism in DSR or RSR. (g) Quantitative analysis of metabolites in the glutamine metabolism pathway and differential expression of metabolic genes. Red indicates upregulated genes, and blue indicates downregulated genes. (h) Relative expression of GLS2 mRNA and protein in DSR and RSR from RAW264.7 cells. (i,j) ELISA results showing the glutamine consumption and glutamate concentration in CM from R, DSR or RSR, respectively. (k) Quantitative analysis results of glutamate content in the supernatant after using C‐IN‐1 to intervene in senescent macrophages (DSR, RSR). Statistical significance was determined by one‐way ANOVA with Tukey's post hoc test (g, h, i, k).

Metabolic reprogramming is a key adaptive mechanism that enables cells to withstand oxidative stress. To identify metabolic alterations in senescent macrophages, we performed metabonomics profiling and identified 20 Kyoto Encyclopedia of Genes and Genomes (KEGG) pathways in cisplatin‐ or radiation‐induced senescent macrophages (Figure S4a,b). Eleven pathways overlapped between the two models, including central carbon metabolism in cancer, aminoacyl‐tRNA biosynthesis, and alanine, aspartate and glutamate metabolism (Figure S4c,d). Sankey analyses revealed 41 and 33 altered metabolites within these shared pathways in cisplatin‐ and radiation‐induced senescent macrophages, respectively (Figure 3a,b). Further, we used a Venn diagram analysis to illustrate that there were 28 differentially abundant metabolites including L‐glutamine and L‐glutamic acid (Figure 3c,d).

To determine gene‐level regulators of these altered metabolites, we performed RNA sequencing (RNA‐seq). Analysis using a fold change ≥ 2 identified 4628 commonly dysregulated genes across both senescent macrophages (Figure S5c,e). Additionally, MetScape network analysis of differentially abundant metabolites positioned glutamine–glutamate metabolism as the central metabolic hub and identified 41 associated core metabolic genes (Figure 3e). Venn comparison between transcriptomic and metabolomic datasets identified overlapping hub genes (Figure 3f). By prioritizing glutamine‐regulatory genes using log_2_(fold change) and network degree, we identified GLS2 as the most significantly upregulated regulator of glutaminolysis, catalyzing the conversion of glutamine to glutamate (Figure 3g).

Consistent with these findings, senescent macrophages showed increased glutamine consumption, elevated glutamate levels, and heightened GLS2 expression (Figure 3h–j; Figure S4j,l,m). Notably, pharmacological inhibition of GLS2 in senescent macrophages using C‐IN‐1 significantly reduced glutamate production compared to untreated controls (Figure 3k; Figure S4k). Glucose consumption and lactate production were reduced, whereas adenosine triphosphate levels remained unchanged (Figure S4g–i). Senescent macrophages also exhibited decreased activity of specific TCA cycle enzymes but increased glutamine influx, supporting the accumulation of key metabolic intermediates (Figure 3g). Gene set enrichment analysis (GSEA) of RNA‐seq data further revealed enrichment of senescence‐associated gene programs following cisplatin or radiation exposure, consistent with metabolic dysregulation (Figure S5d). Together, these results indicate cisplatin‐ and radiation‐induced senescent macrophages exhibit active glutamine metabolism.

### 2.4 Glutamine Metabolic Reprogramming Drives IL‐1β Secretion in Senescent Macrophages

**FIGURE 4:**
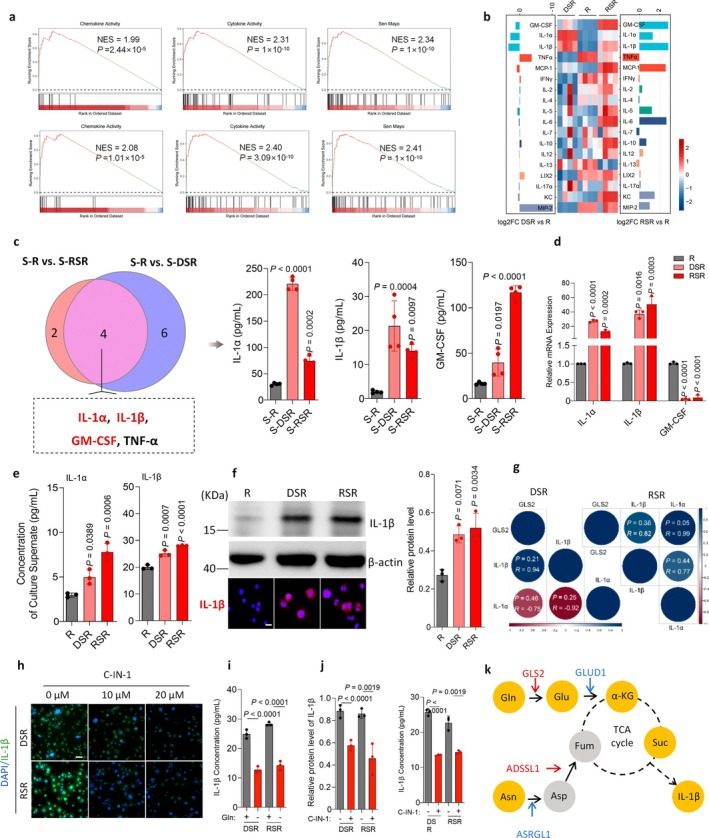
Glutamine metabolism in senescent macrophages drives the synthesis and secretion of IL‐1β. (a) GSEA results of senescent macrophages (DSR and RSR) in chemokine activity, cytokine activity, Sen Mayo sets. (b) Heat map in 18 cytokines of supernatants from direct co‐culture of SCC7 with macrophages (S‐R, S‐DSR or S‐RSR) assessed by the MILLIPLEX assay. (c) Venn diagram showing 4 differentially expressed cytokines including GM‐CSF, IL‐1α, IL‐1β, and TNF‐α in the supernatants. (d) Relative mRNA expression of GM‐CSF, IL‐1α, and IL‐1β in the DSR or RSR RAW264.7 cells. (e) ELISA determination of increased IL‐1β levels in the supernatants of DSR and RSR. (f) Immunoblots and representative immunofluorescence images of IL‐1β in the intracellular protein of R, DSR, and RSR. (g) Pearson correlation analysis showing a significant positive correlation between GLS2 and IL‐1α/β. (h) Representative immunofluorescence images of IL‐1β in DSR or RSR treated with different concentrations of C‐IN‐1. Scale bars, 50 μm. (i) IL‐1β expression analysis under different glutamine‐deprivation conditions in DSR or RSR. (j) Immunoblots and ELISA results showing IL‐1β levels in DSR or RSR treated with 20 μM C‐IN‐1. (k) KEGG pathway analysis of altered gene and metabolites in senescent macrophages. Increased glutamine metabolism in senescent macrophages was involved in central carbon metabolism through entry into the tricarboxylic acid cycle (TCA cycle): Yellow dots, increased metabolites; gray dots, no significantly changed metabolites; red arrows, increased genes; blue arrows, decreased genes. *n* = 3 in each group from independent biological replicates (d, e, f, i, j), *n* = 4 in each group from independent biological replicates (c). Statistical significance was determined by one‐way ANOVA with Tukey's post hoc test; mean ± s.d. (c, d, e, f, i, j).

GSEA of RNA‐seq data showed marked enrichment of chemokine activity, cytokine activity, and SenMayo gene sets in senescent macrophages (Figure 4a). To validate these transcriptomic findings, we used a MILLIPLEX assay to assess SASP secretion. Heatmap analysis revealed distinct secretome profiles in supernatants from the co‐culture model (Figure 4b). IL‐1α, IL‐1β, and granulocyte‐macrophage colony‐stimulating factor (GM‐CSF) were significantly upregulated, whereas TNF‐α was significantly downregulated in senescent macrophages (Figure 4c). Given the known myelopoietic role of GM‐CSF and the established suppression of TNF‐α in senescent macrophages (Guerrero et al. 2024), we focused on IL‐1α and IL‐1β as potential dominant SASP effectors.

qRT‐PCR analysis confirmed increased IL‐1α and IL‐1β mRNA, with reduced GM‐CSF in senescent macrophages supernatants (Figure 4d; Figure S7k). Although immunofluorescence staining showed elevated intracellular IL‐1α and IL‐1β (Figure 4f; Figure S7L), extracellular IL‐1α showed elevated intracellular in conditioned medium (CM) from cisplatin‐induced J774A.1 senescent macrophages (Figure 4e; Figure S7m). Consistent with the previous data, IL‐1β increased significantly in both extracellular and intracellular protein levels by ELISA, WB, and immunofluorescence assays (Figure 4e,f; Figure S7m,n). Thus, IL‐1α accumulation occurred intracellularly without active secretion. Consistent with the previous data, IL‐1β increased significantly in both extracellular and intracellular protein levels by ELISA, WB, and immunofluorescence assays (Figure 4e,f; Figure S7m,n). These results establish IL‐1β as a key SASP effector in senescent macrophages.

Next, we assessed correlation among these genes using Pearson correlation coefficient and identified a statistically significant positive association between GLS2 and IL‐1β (Figure 4g). To explore the effects of upregulated GLS2 on IL‐1β, we removed exogenous glutamine from the senescent macrophages and observed a significant reduction in IL‐1β levels (Figure 4i; Figure S5k). Because GLS2 catalyzes the conversion of glutamine to glutamate, we further inhibited glutamine metabolism with the GLS‐specific inhibitor C‐IN‐1; cellular immunofluorescence staining and WB analysis showed that C‐IN‐1 substantially suppressed IL‐1β expression in senescent macrophages (Figure 4h,j; Figure S5g–j). Together, these findings suggest that glutamine availability and GLS2 activity drive IL‐1β expression in senescent macrophages and that this process is reversible through GLS inhibition (Figure 4k).

### 2.5 Senescent Macrophages Accelerate SCC Invasion by a SASP

To investigate how the senescent macrophage secretome influences SCC progression, we examined biological responses in SCC‐7 cells. CM from cisplatin‐ or radiation‐induced senescent macrophages significantly reduced SCC‐7 cell proliferation, accompanied by a decrease in G2/S‐phase cells as shown by CCK‐8 and flow cytometry assays (Figure S6a,e–g). Colony formation assays demonstrated that senescent macrophages' CM significantly reduced SCC‐7 colony numbers and size (Figure S6b). Notably, SCC‐7 cells remained SA‐β‐gal negative and showed no substantial changes in senescence‐related protein expression following CM or transwell exposure (Figure S6c,d). These results indicate that senescent macrophages suppress SCC‐7 proliferation in vitro through paracrine mechanisms but do not transmit senescence programs.

**FIGURE 5:**
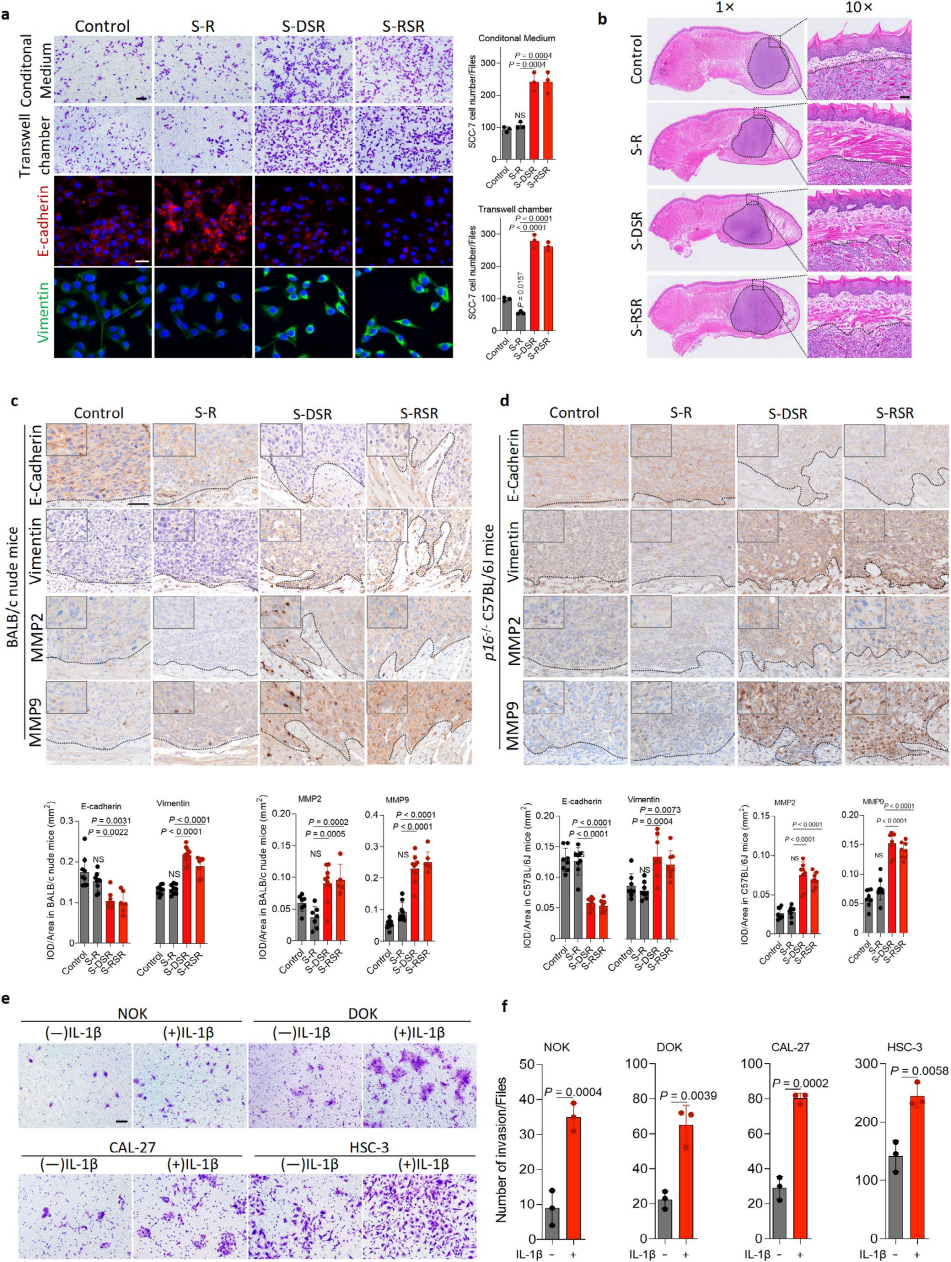
Senescent macrophages secrete IL‐1β for promoting oral cancer invasion. (a) Top: Representative images and quantification of invasion in transwell assays using conditioned medium (CM) or co‐culture with R, DSR or RSR. SCC‐7 cells were treated with CM derived from R, DSR or RSR. Bottom: Representative immunofluorescence images of E‐cadherin and Vimentin in SCC‐7 cells co‐cultured in transwell chamber under different conditions (alone, R, DSR, RSR, J, DSJ or RSJ). Scale bar, 100 μm (top) and 50 μm (bottom). (b) H&E staining images of tongue orthotopic OSCC xenograft model. Scale bar, 50 μm. (c‐d) Representative IHC images and quantification analysis of E‐cadherin, Vimentin, MMP2 and MMP9 staining in tumor sections from BALB/c nude mice and *p16* ^−/−^ C57BL/6J mice implanted with SCC‐7 cells with or without macrophages. BALB/c nude mice model: SCC‐7 cells alone (*n* = 9), SCC‐7 cells and RAW264.7 cells (S‐R) (*n* = 9), SCC‐7 cells and DSR (S‐DSR) (*n* = 10), SCC‐7 cells and RSR (S‐RSR) (*n* = 7). *p16* ^−/−^ C57BL/6J mice model: SCC‐7 cells (*n* = 8), S‐R (*n* = 8), S‐DSR (*n* = 8), and S‐RSR (*n* = 8). Scale bar, 50 μm. (e‐f) Representative images and quantification of invasion assays. Human derived cell lines including normal oral keratinocytes (NOK), dysplastic oral keratinocytes (DOK), tongue squamous cell carcinoma cells (CAL‐27) and OSCC cells (HSC‐3) were treated with exogenous IL‐1β. Scale bar, 100 μm. *n* = 3 in each group from independent biological replicates (a, e). Statistical significance was determined by one‐way ANOVA with Tukey's post hoc test; mean ± s.d. (a, c, d, f).

We then evaluated the impact of senescent macrophages on SCC invasion. CM or transwell co‐culture with senescent macrophages significantly enhanced SCC‐7 invasion and migration (Figure 5a; Figure S6h–k). Similarly, scratch and transwell assays demonstrated that senescent macrophage CM or co‐culture accelerated wound closure within 18 h (Figure S7a–c). As Vimentin and E‐cadherin expression changes are frequently observed in cancer cells and are correlated with increased invasiveness (Tian et al. 2020), we assessed these markers by immunofluorescence staining and found that SCC‐7 cells co‐cultured with senescent macrophages exhibited elevated Vimentin and reduced E‐cadherin expression relative to controls (Figure 5c; Figure S7d).

Moreover, to determine whether senescent macrophages consistently promote EMT‐mediated invasion in vivo, we generated SCC models in immunodeficient nude mice and *Cdkn2a* ^*−*/−^ C57BL/6 mice by co‐injecting SCC‐7 cells with cisplatin‐ or radiation‐induced senescent macrophages (Figure S7e–g). Our data showed that tumors from mice co‐injected with SCC‐7 cells and senescent macrophages exhibited upregulation of Vimentin, MMP‐2, and MMP‐9, and downregulation of E‐cadherin (Figure 5b–d; Figure S7i–j). To evaluate whether IL‐1β is required for SCC invasion, we treated cells with recombinant IL‐1β in transwell assays and found that it significantly enhanced migration and invasion in normal human keratinocytes (NOK), premalignant cells (dysplastic oral keratinocyte [DOK]), and malignant oral epithelial cells (CAL‐27 and HSC‐3) (Figure 5e,f; Figure S8a). Recombinant IL‐1β significantly accelerated scratch wound closure across oral epithelial cell models: with DOK and CAL‐27 responding by 24 h, NOK by 48 h, and metastatic HSC‐3 by 10 h (Figure S8b–d). Together, these results demonstrate that senescent macrophages drive SCC invasion by inducing EMT activation through paracrine signaling, promoting mesenchymal reprogramming from 2D models to immunocompetent (*Cdkn2a* ^−/−^) and immunodeficient xenograft systems.

### 2.6 Senescent Macrophage‐Derived IL‐1β Drives SCC Invasion Through IL‐1R2/NF‐κB Signaling

**FIGURE 6:**
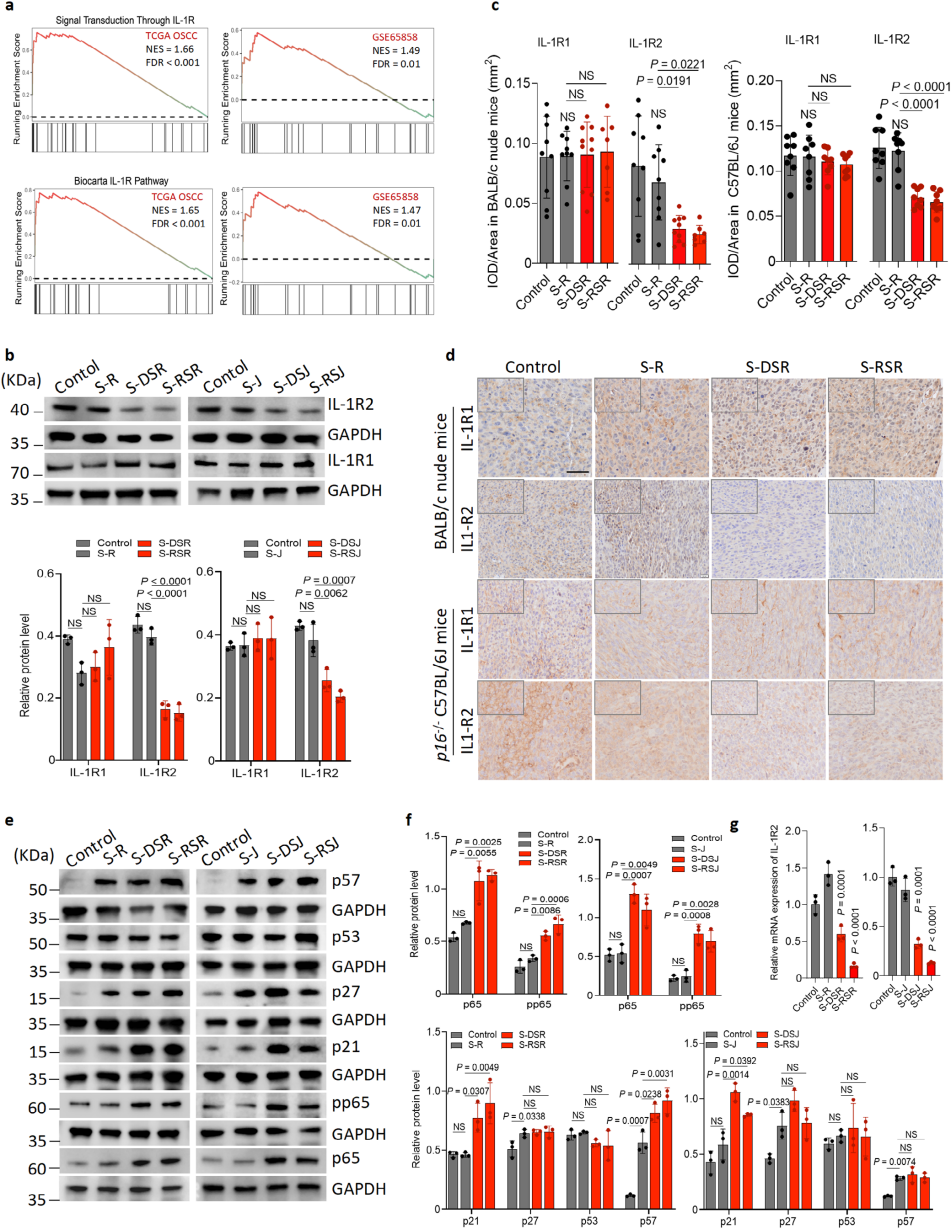
Senescent macrophage‐derived IL‐1β drives oral cancer invasion via the IL‐1β/IL‐1R/NFκB signaling cascade. (a) Gene Set Enrichment Analysis (GSEA) results of OSCC tissues from the TCGA and GSE65858 datasets in Signal Transduction Through IL‐1R and Biocarta IL‐1R pathway sets. (b, c) Immunoblots and quantification of IL‐1R1 and IL‐1R2 levels in SCC‐7 cells indirectly co‐cultured with R, DSR, RSR, J774A.1 cells (J), DSJ or RSJ. (d) Representative IHC images of IL‐1R1 and IL‐1R2 staining in tumor sections from BALB/c nude mice and *p16* ^−/−^ C57BL/6J mice implanted with SCC‐7 cells with/without macrophages. BALB/c nude mice model: SCC‐7 cells (*n* = 9), S‐R (*n* = 9), S‐DSR (*n* = 10), S‐RSR (*n* = 7). *p16* ^−/−^ C57BL/6J mice model: SCC‐7 cells (*n* = 8), S‐R (*n* = 8), S‐DSR (*n* = 8), and S‐RSR (*n* = 8). Scale bar, 50 μm. (e, f). Immunoblots and quantification of p21, p27, p53, p57, p65, and pp65 levels in SCC‐7 cells indirectly co‐cultured with R, DSR, RSR, J, DSJ or RSJ. (g) Relative expression of IL‐1R2 mRNA in senescent macrophage. *n* = 3 in each group from independent biological replicates and statistical significance was determined by one‐way ANOVA with Tukey's post hoc test; mean ± s.d. (b, f).

Next, we explored the mechanism by which IL‐1β enhanced OSCC invasion. To investigate potential target molecules, we applied the GSEA algorithm and identified gene sets and downstream signals associated with the IL‐1 receptor (IL‐1R) pathway in OSCC cells (Figure 6a). We further assessed IL‐1 receptor expression, including IL‐1R1 and IL‐1R2, by Western blot. The results showed a significant downregulation of IL‐1R2, with no obvious change in IL‐1R1, in SCC‐7 cells incubated with senescent macrophages. Consistently, qRT‐PCR analysis demonstrated that IL‐1R2 mRNA expression was also significantly decreased under the same conditions (Figure 6b,g). Consistently, in two xenograft models, tumors originating from SCC‐7 cells and senescent macrophages exhibited markedly reduced IL‐1R2 expression, while IL‐1R1 remained unchanged (Figure 6c,d). These findings suggest that senescent macrophage‐derived IL‐1β promotes SCC invasion through IL‐1R2‐dependent signaling derepression.

Given that nuclear factor‐kappaB (NF‐κB) in cancer cells is closely associated with cellular invasion, and our results indicated enhanced SCC‐7 invasion induced by senescent macrophage‐derived IL‐1β (Kaur et al. 2023), a likely link exists between the NF‐κB signaling cascade and senescent macrophage‐driven SCC invasion. To test this, we performed WB and observed significant upregulation of the major NF‐κB subunit p65 when SCC‐7 cells were indirectly co‐cultured with senescent macrophages (Figure 6e,f). Next, we examined activation of the NF‐κB pathway, particularly p65 phosphorylation (pp65), and found increased pp65 levels in indirectly co‐cultured SCC‐7 cells (Figure 6e,f). These findings indicate that NF‐κB signaling may mediate the invasive response of cancer cells to senescent macrophages.

Notably, although NF‐κB signaling typically promotes proliferation in cancers (Kumar et al. 2021), we demonstrated that its activation in senescent macrophages suppressed proliferation while enhancing invasiveness, suggesting that these functions are uncoupled. Because cyclin‐dependent kinases are critical for cell proliferation and their inhibitors (CKIs) including p21, p27, and p57 suppress this process (Zhang et al. 2021), we assessed CKI expression of CKIs in SCC‐7 cells. Our data showed that p21 increased significantly in SCC‐7 cells indirectly co‐cultured with senescent macrophages, whereas p27 and p57 did not exhibit consistent increases (Figure 6e,f). Furthermore, no significant change in p53 expression was detected in co‐cultured SCC‐7 cells (Figure 6e,f).

Collectively, these results suggest that senescent macrophage‐derived IL‐1β promotes SCC invasion through IL‐1R2/NF‐κB signaling via p65 phosphorylation while suppressing cell proliferation through an IL‐1R2/p21 signaling cascade.

### 2.7 IL‐1β Blockage Attenuates SCC Invasion Induced by Senescent Macrophages

**FIGURE 7:**
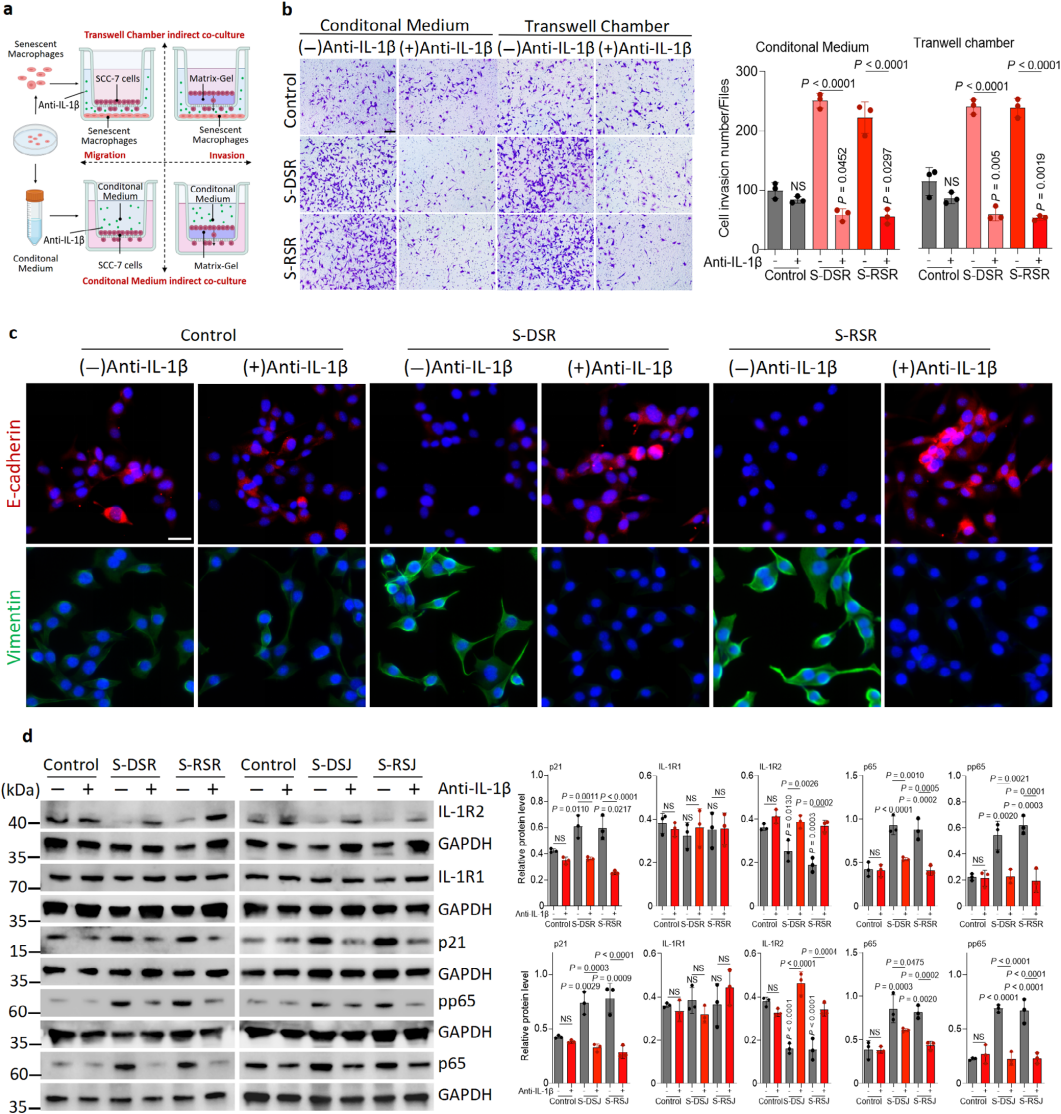
Exogenous recombinant IL‐1β promotes oral cancer invasion and blocking IL‐1β attenuates this process and downstream signaling pathway. (a) Schematic of the migration and invasion transwell assays. Briefly, SCC‐7 cells in a transwell chamber were challenged to migrate through transwell with 5 μm pores. For the invasion assay, SCC‐7 cells were seeded at the bottom of the transwell chambers that had been coated with matrigel. The migration or invasion of SCC‐7 cells was induced by senescent macrophage‐derived CM or indirect co‐culture with senescent macrophages. (b) Representative images and quantification of invasion assays after treatment with an anti‐IL‐1β antibody. Scale bar, 100 μm. (c) Representative images of immunofluorescence staining of E‐cadherin and Vimentin in SCC‐7 indirectly co‐cultured with DSR or RSR, treated with or without anti‐IL‐1β antibody. Scale bars, 100 μm. (d) SCC‐7 cells indirectly co‐cultured with senescent macrophages were treated with an anti‐IL‐1β antibody. Immunoblots of p21, IL‐1R1, IL‐1R2, p65, and pp65 levels in SCC‐7 cells. *n* = 3 in each group from independent biological replicates and statistical significance was determined by one‐way ANOVA with Tukey's post hoc test; mean ± s.d. (b, d).

To identify senescent macrophage‐derived IL‐1β in SCC invasion, we performed transwell assays using a neutralizing antibody (Figure 7a). Anti‐IL‐1β antibody treatment significantly reduced the invasive and migrative abilities of SCC‐7 cells induced by senescent macrophage‐derived CM or the indirect co‐culture system (Figure 7b; Figure S8e,f,h). Together, these findings demonstrated that senescent macrophage‐derived IL‐1β is a key driver of SCC invasion, and that blocking IL‐1β effectively suppresses this pro‐invasive response.

We next investigated the mechanism by which senescent macrophage‐derived IL‐1β promoted SCC invasion. Immunofluorescence staining showed that anti‐IL‐1β antibody treatment increased E‐cadherin expression while reducing Vimentin levels in SCC‐7 cells indirectly co‐cultured with senescent macrophages (Figure 7c; Figure S8g). As described above, senescent macrophage‐derived IL‐1β upregulated NF‐κB and p21, and downregulated IL‐1R2. Therefore, we hypothesized that anti‐IL‐1β might reverse these alterations. As expected, WB demonstrated that the antibody significantly reduced p21, p65, and pp65 levels in SCC‐7 cells exposed to senescent macrophages (Figure 7d). Notably, anti‐IL‐1β antibody substantially increased IL‐1R2 expression in SCC‐7 cells while exerting no effect on IL‐1R1 level (Figure 7d). In sum, these results indicate that IL‐1β blockade reverses senescent macrophage‐driven EMT and NF‐κB/p21 activation in SCC by restoring IL‐1R2, therefore leading to reduced SCC invasion.

### 2.8 Blocking IL‐1β Restores Epithelial Traits and Reduces the Invasive Behavior of SCC In Vivo

**FIGURE 8:**
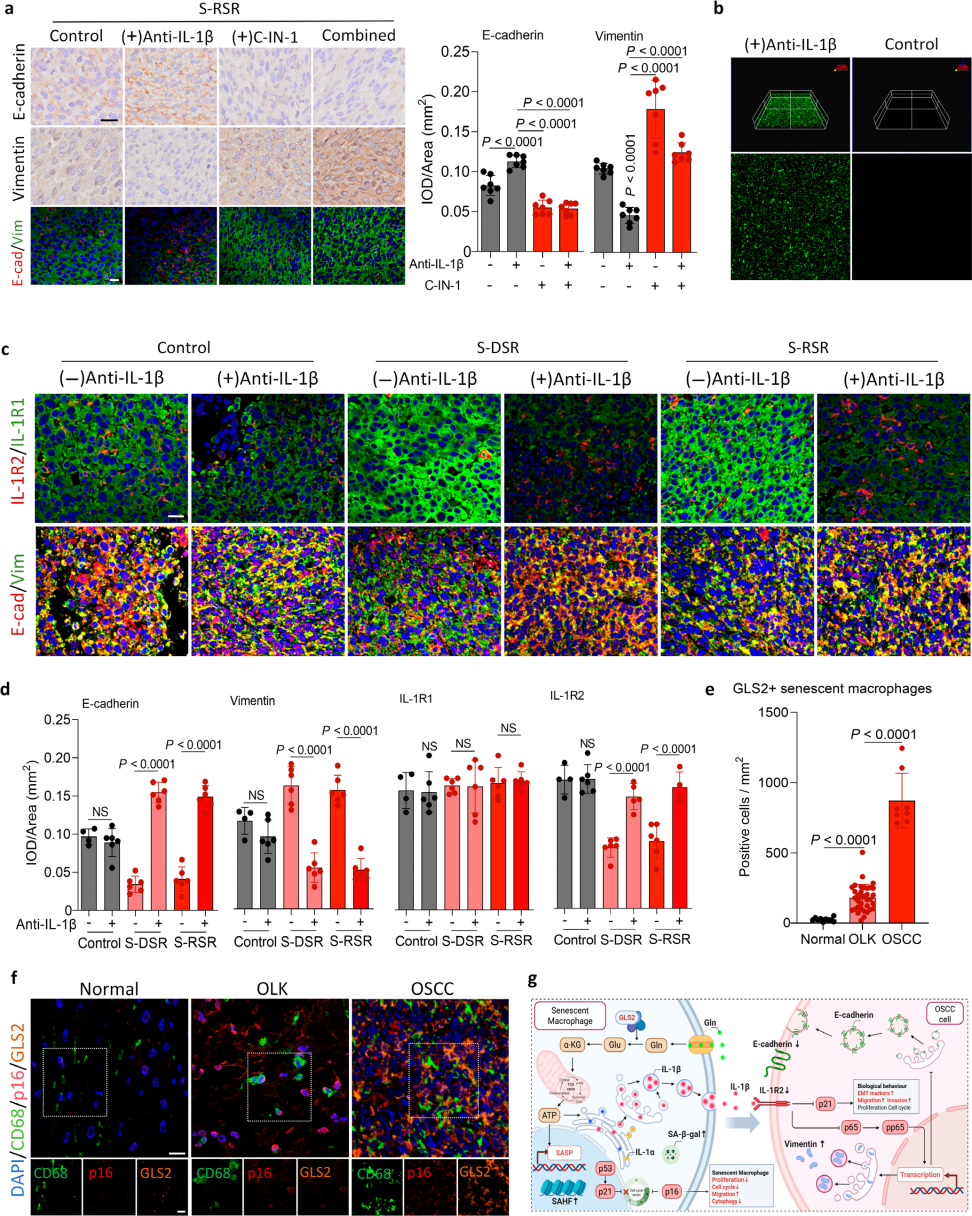
Inhibition of IL‐1β reduces Vimentin and increases E‐cadherin to suppress OSCC invasion in vivo. (a) Representative IHC and mIHC images, and quantification of E‐cadherin and Vimentin in tumor sections from S‐RSR treated with anti‐IL‐1β antibody, C‐IN‐1, or a combination of the anti‐IL‐1β antibody and C‐IN‐1 (*n* = 7 independent biological replicates for each group). Scale bars, 20 μm (upper), 20 μm (bottom). (b) Representative images showing anti‐IL‐1β antibody in 15% w/v PLGA‐PEG‐PLGA gel. (c, d) Representative mIHC images (c) and quantification (d) of E‐cadherin, Vimentin, IL‐1R1 and IL‐1R2, in nude mice bearing tumor derived from SCC‐7 cells alone, S‐DSR or S‐RSR treated with or without the anti‐IL‐1β antibody (*n* = 6 in the comparisons except *n* = 4 in the group of SCC‐7 cells alone + vehicle). Scale bars, 20 μm. (e, f) Representative mIHC images (f) and quantification (e) of CD68, p16, and GLS2 in normal human oral mucosa, OLK and OSCC tissues (*n* = 10 in normal, *n* = 38 in OLK, *n* = 8 in OSCC). Scale bars, 20 μm (upper), 10 μm (bottom). (g) Schematic illustrating GLS2+ senescent macrophages with activated glutamine metabolism promote Vimentin expression and suppress E‐cadherin during OSCC invasion. The glutamine metabolism/IL‐1β/IL‐1R/NFκB pathway is critical for this process, but it can be inhibited by blocking IL‐1β. Statistical significance was determined by one‐way ANOVA with Tukey's post hoc test; mean ± s.d. (a, d, e).

The above findings showed that GLS2 markedly increased IL‐1β secretion in senescent macrophages. To further validate the contribution of senescent macrophage‐derived IL‐1β to SCC invasion, we established a nude mouse subcutaneous model in which SCC‐7 cells were transplanted with or without senescent macrophages. Intriguingly, unlike the suppressive effect of the anti‐IL‐1β antibody on SCC invasion, C‐IN‐1 treatment, either alone or combined with the anti‐IL‐1β, decreased E‐cadherin expression and increased Vimentin levels in tumor tissues (Figure 8a). Likewise, C‐IN‐1 alone or combined with anti‐IL‐1β significantly enhanced SCC‐7 cell invasion and migration (Figure S9d). Because C‐IN‐1 inhibited glutamine metabolism, we questioned how tumor cells obtained energy to sustain invasive behaviors. IHC staining showed that C‐IN‐1 activated HIF‐1α and PKM2, thereby promoting glycolysis and fueling tumor progression (Figure S9f).

To enhance the therapeutic effect of an exogenous IL‐1β inhibitor in vivo, we loaded the anti‐IL‐1β antibody into 15% w/v PLGA‐PEG‐PLGA for treatment (Figure 8b). We injected the anti‐IL‐1β antibody gel into the nude mice bearing tumors originating from SCC‐7 cells and senescent macrophages, and observed that the gel increased E‐cadherin expression while significantly reducing Vimentin levels compared with the controls (Figure S9h). Injection of the anti‐IL‐1β antibody gel also markedly increased IL‐1R2 expression in tumors derived from SCC‐7 cells and senescent macrophages, with no evident change in IL‐1R1 (Figure 8c,d; Figure S9h). In contrast, in controls, the antibody gel did not alter E‐cadherin, Vimentin, IL‐1R1 or IL‐1R2 in tumors originating from SCC‐7 cells alone (Figure 8c,d: Figure S9h). Tumor weight showed no significant differences among groups treated with the anti‐IL‐1β antibody gel (Figure S9g). In a clinical context, mIHC staining demonstrated significantly increased infiltration of CD68^+^/p16^INK4a+^/GLS2^+^ senescent macrophages across human oral normal mucosa, OLK, and OSCC tissues (Figure 8e,f). Overall, these findings suggest that senescent macrophage‐derived IL‐1β promotes SCC invasion through the IL‐1β/IL‐1R2 pathway, and that targeting this axis with anti‐IL‐1β antibody delivered via PLGA‐PEG‐PLGA can substantially inhibit SCC invasion.

## 3 Discussion

Globally, > 90% of patients with cancer succumb to metastasis, and available therapeutic options remain limited because conventional treatments struggle to halt the metastatic progression in cancers (Gerstberger et al. 2023). As cellular invasion is a central hallmark of cancer metastasis (Hanahan 2022), there is an urgent need to identify novel anti‐invasive approaches to improve treatment outcomes (Yang et al. 2026; Kwa et al. 2026). In this study, we found that senescent macrophages exhibit enhanced GLS2‐driven glutamine metabolism, and their upregulated IL‐1β promotes SCC cell invasion in an IL‐1R2‐dependent manner. Targeting senescent macrophage‐derived IL‐1β reduced Vimentin expression and increased E‐cadherin expression, thereby suppressing the EMT process. These findings underscore the potential promotive role of senescent macrophages in SCC invasion.

The concept of “oxidative stress” in redox biology, first proposed by Sies in 1985, describes disrupted redox homeostasis caused by an imbalance in ROS generation and elimination (Sies 2015). As research has advanced, oxidative stress is no longer viewed solely as a damaging state but as a perturbation of redox signaling. Moderate ROS levels act as signaling mediators in physiological regulation, whereas excessive ROS accumulation leads to cellular dysfunction and disease (Jones 2006; Ray et al. 2012; Reybrouck 2014). Here, we demonstrated that cisplatin or radiation, classical inducers of oxidative stress in tumors, can drive macrophages into durable cell cycle arrest, and that these SA‐β‐Gal^+^ senescent macrophages display unique biological behaviors. As a universal feature of cellular senescence, the SASP promotes inflammation, infections, and malignancies through proinflammatory cytokines, CXC chemokine ligands, and extracellular matrix proteases (Mosteiro et al. 2016). Consistent with previous reports, we observed that oxidative stress–induced senescent macrophages exhibit high IL‐1β secretion. SASP has indeed been identified as an early marker of cellular senescence (Coppé et al. 2008; Young and Narita 2009). Our data further showed that GLS2 enhances IL‐1β secretion via endogenous glutamine metabolism, and that inhibiting GLS2 significantly reduces IL‐1β release in senescent macrophages. These results indicate that IL‐1β expression is upregulated by GLS2‐mediated glutamine metabolism and that GLS2 activity represents an early event in the secretory phenotype of senescent macrophages.

Glutamine and glucose are the two major consumable nutrients for tumor cells, supporting energy metabolism, anabolic activity, and redox homeostasis, respectively (Wise and Thompson 2010; Altman et al. 2016). Under oxidative stress, glutamine assumes additional roles linked to its function as a carbon–nitrogen hub. As a precursor of GSH and a promotor of NADPH generation, it removes ROS and maintains redox balance, thereby providing tumor cells with survival and invasive advantages during elevated oxidative stress (Gong et al. 2022; Fan et al. 2024). Consequently, certain tumor cells develop glutamine addiction, a hallmark of metabolic reprogramming in cancer (Hensley et al. 2013). Glutamine addiction indicates that tumor cells remain dependent on glutamine despite abundant nutrient availability (Wise and Thompson 2010). During tumor invasion and metastasis, ROS act as signaling molecules that activate EMT (Radisky et al. 2005; Pan et al. 2012); however, excessive ROS cause cell death; tumor cells therefore rely on glutamine metabolism to buffer ROS via NADPH and GSH production, sustaining invasion and metastasis under moderate ROS conditions (Hensley et al. 2013; Trachootham et al. 2009; Son et al. 2013). According to Zou et al., glutamine metabolism maintains redox homeostasis through these mechanisms, forming a metabolic foundation for glutamine addiction in aggressive tumors (Zou et al. 2025).

In our study, oxidative stress–induced senescent macrophages exhibited elevated glutamine metabolism, which facilitated SCC invasion. This finding suggests that glutamine metabolism may fuel this invasive process and raises the possibility that SCC is glutamine addicted, offering a potential direction for metabolic targeting therapy. Among therapeutic strategies for glutamine‐addicted tumors, GLS inhibition remains a well‐established approach. Telaglenastat (CB‐839), a representative glutaminase inhibitor, has undergone Phase I/II cancer trials and is well tolerated by patients (Wicker et al. 2021). However, the CANTATA randomized clinical trial reported that combining CB‐839 with cabozantinib in patients with mRCC did not improve progression‐free survival or overall survival (Tannir et al. 2022). Our data also demonstrated that pharmacological IL‐1β inhibition, when combined with GLS2 blockade, produced a significant invasive promotion, suggesting that GLS2‐mediated glutamine metabolism and/or its metabolites may directly suppress invasiveness. Similar paradoxical roles for GLS2 have been reported in breast and liver cancers (Suzuki et al. 2022; Dias et al. 2020), reinforcing the view that GLS2 functions are cell‐ and tissue‐specific.

These findings suggest that GLS2 inhibition may not effectively suppress tumor progression due to metabolic plasticity and context‐dependent responses. In contrast, targeting downstream inflammatory mediators such as IL‐1β might bypass these adaptive mechanisms and provide a more stable and direct anti‐invasive effect. Therefore, rather than representing a contradiction, our results underscore the complexity of metabolic‐inflammatory crosstalk and highlight the importance of selecting therapeutically actionable nodes within this regulatory network. Similarly, Opitz et al. found that therapeutically targeting aryl hydrocarbon receptor, which is the critical downstream receptor of kynurenine pathway signaling, could counteract the compensatory effect of indoleamine 2,3‐dioxygenase/tryptophan 2,3‐dioxygenase in tryptophan‐mediated tumor immunosuppression (Opitz et al. 2011), demonstrating that therapeutically targetable pathways do not necessarily represent primary mechanistic drivers.

As a key proinflammatory cytokine, IL‐1β exerts its biological functions by binding to its downstream high‐affinity receptors, particularly IL‐1R1 and IL‐1R2 (van Baarle et al. 2024). IL‐1R2 known as a decoy receptor, competitively binds and sequesters IL‐1β but lacks the intracellular toll/interleukin‐1 receptor (TIR) domain required for IL‐1β signal transduction (Gaballa et al. 2024). Our in vitro and in vivo findings showed that senescent macrophages downregulated IL‐1R2 expression in head and neck squamous cell carcinoma (HNSCC) cells without affecting IL‐1R1 levels. Consistent with this observation, injection of an IL‐1β inhibitor gel significantly increased IL‐1R2 expression in mice bearing HNSCC tumors derived from SCC‐7 cells and senescent macrophages, whereas IL‐1R1 levels remained unchanged. These findings suggest that senescent macrophage‐derived IL‐1β reduces IL‐1R2 expression and activates the IL‐R1 signaling cascade in cancer cells. Intriguingly, our results demonstrated that senescent macrophages strongly promoted cellular invasion while suppressing tumor cell proliferation. In contrast, senescent alveolar macrophages have been reported to enhance Ki‐67 expression and malignant progression by creating an immunosuppressive microenvironment in premalignant lung tumors (Haston et al. 2023). In our study, cisplatin‐ or radiation‐induced senescent macrophages increased p21 expression in cancer cells, thereby blocking the transition from G1 to G2/S and inhibiting proliferation via the IL‐1β/IL‐1R2/p21 signaling cascade. These findings indicate that senescent macrophages can act as tumor‐initiating events yet exhibit paradoxical roles in regulating proliferation. Notably, pathways controlling proliferation and invasion are not always linked. IL‐1β binds IL‐1R1, leading to NF‐κB activation that promotes cancer invasion (Yang et al. 2026). Supporting this mechanism, we analyzed the NF‐κB signaling state of p65 and pp65 in cytoplasmic fractions and found that senescent macrophage‐derived IL‐1β increased both p65 and pp65 expression, thereby promoting SCC invasion through the IL‐1β/IL‐1R2/NF‐κB pathway. This raises the question of how cancer cells meet the energy demands required for invasion. Due to aerobic glycolysis, or the Warburg effect, cancer cells increasingly rely on glycolysis rather than oxidative phosphorylation to satisfy energy needs (Fendt 2024). As expected, we found that inhibiting glutamine metabolism with C‐IN‐1 activated aerobic glycolysis as a positive feedback mechanism supporting aggressive oral cancer.

In addition to the mechanistic insights described above, it is important to consider the experimental context in which these findings were obtained. In this study, *p16* ^*−/−*^ mice were used to establish a host environment with a reduced endogenous senescent cell burden, thereby minimizing background interference and enabling a clearer evaluation of the effects of exogenously introduced senescent macrophages. Importantly, all experimental groups were maintained within the same p16‐deficient background, ensuring that systemic effects of p16 loss were consistent across groups. As SCC‐7 tumor cells were exogenously implanted and do not carry p16 deficiency, the observed phenotypes are more likely attributable to the functional impact of senescent macrophages rather than direct alterations in tumor cell‐intrinsic cell cycle regulation. Nevertheless, it should be noted that the use of global p16−/− mice may introduce systemic effects across multiple cell types, which could influence the tumor microenvironment. Within this framework, our findings further support a central role for SASP‐mediated signaling in regulating tumor progression.

It is important to note that the SASP is regarded as the most prominent feature of cellular senescence, and the NF‐κB pathway serves as the major regulatory mechanism controlling SASP production (Strzeszewska et al. 2018; Luo et al. 2025). Mechanistically, mTOR can upregulate IL‐1α expression, thereby activating NF‐κB signaling to promote translation of SASP mRNAs (Orjalo et al. 2009). Our findings also demonstrated that the NF‐κB cascade in cancer cells functions as a downstream effector activated by SASP within the surrounding microenvironment. Rapamycin exerts significant anti‐SASP effects, and its combination with mTOR inhibitors can reverse senescent transition in presenescent cells (Laberge et al. 2015; Walters et al. 2016). However, rapamycin use in clinical settings may cause adverse effects, including immunosuppression, impaired glucose tolerance, and lipid abnormalities (Blättler et al. 2012; Trelinska et al. 2015). To this end, the compound DL001, as a novel mTOR inhibitor, has shown markedly greater selectivity toward mTORC1 than rapamycin (Schreiber et al. 2019). Given that the dual tumor‐modulatory roles of senescent cells highly depend heavily on SASP heterogeneity, preserving tumor‐suppressive SASP components while inhibiting tumor‐promotive SASP remains essential. Therefore, elucidating the signaling pathways that regulate tumor‐promotive SASP and developing strategies to block these pathways are urgently needed.

Collectively, our work highlights the critical role of senescent macrophages in promoting cancer invasion through glutamine metabolism. We identify a mechanism by which GLS2‐dependent IL‐1β secretion from senescent macrophages influences glutamine metabolism in cancer cells in an IL‐1R2‐dependent manner. Importantly, targeting IL‐1β was more effective in suppressing cancer invasion than GLS2 inhibition. These findings reveal a previously unrecognized metabolism linking glutamine metabolism and the SASP through senescent macrophages and propose a potential metabolic intervention strategy to counteract SCC invasion.

## Author Contributions

H.Z., F.W., and J.L. contributed to the conception, and designed the experiments and supervised the project. S.W., J.M., and J.L. carried out the experiments, analyzed the data and wrote the manuscript. W.Z. and C.H. helped to collect the human samples and performed the experiments. X.S. helped to analyze the data. H.Z., F.W., J.L., S.W., and J.M. discussed and edited paper. All authors approved the manuscript.

## Funding

This study was supported by grants from the National Key Research and Development Program of China 2023YFC3605600 (F.W.), the National Natural Science Foundation of China 82370963 (F.W.), 82573832 (H.Z.), 82301094 (S.W.), and 82301095 (X.S.), and the Sichuan Science and Technology Program 2026NSFSC0673 (F.W.).

## Disclosure

Generative AI and AI‐assisted technologies were not used in the preparation of this work.

## Ethics Statement

This study strictly adhered to all relevant ethical guidelines and regulations. Clinical data and human tissue samples were collected in compliance with the Ethics Committee of the West China Hospital of Stomatology, Sichuan University (No. WCHSIRB‐D‐2019‐012, No. WCHSIRB‐D‐2020‐073, and No. WCHSIRB‐D‐2023‐203).

## Conflicts of Interest

The authors declare no conflicts of interest.

## Supporting information


**Figure S1:** Additional differential infiltrations of senescent macrophages promote oral cancer progression. (a) Representative H&E staining and IHC images of CD68, p16, Ki‐67, p53, E‐cadherin and vimentin staining in human oral tissues. Scale bars, 100 μm for H&E, 50 μm for IHC. (b) Representative mIHC images showing the co‐expression of CD68 and p16 proteins in human oral normal mucosa, OLK, and OSCC patients. Scale bars, 25 μm (left) and 50 μm (right). (c) Co‐expression of CD68, p16 and Ki‐67 in mIHC staining images. Scale bars, 25 μm (upper), 50 μm (bottom). (d) Representative mIHC images showing the co‐expression of CD68, p16, and p53 proteins in human oral normal mucosa, OLK, and OSCC patients. Scale bars, 100 μm (upper), 50 μm (bottom). (e, f) Feature plot (c) and UMAP plot (d) of scRNA‐seq data showing all cells from OSCC tumors with metastasis were identified as 17 groups of cells. (g) GSEA enrichment plot of bulk RNA sequencing of senescence‐associated genes between non‐metastasis OSCC and OSCC with metastasis. Statistical significance was determined by permutation analysis in GOBP Cellular Senescence, FRIDMAN Senescence, and SEN MAYO sets.
**Figure S2:** Additional characterization of cisplatin or radiation induced senescent cells. (a) Schematic of the in vitro experiments for establishing of drug‐induced senescent macrophages and radiation‐induced senescent macrophages. (b, d) J774A.1 cells (J) were treated by cisplatin (drug‐induced senescent J774A.1 cells, DSJ) or radiation (radiation‐induced senescent J774A.1 cells, RSJ) and stained for SA‐β‐Gal (b). Quantitation of percent SA‐β‐Gal^+^ cells (d). Scale bars, 100 μm. (c) Representative fluorescent images of H3K9me in J, DSJ, and RSJ. The accumulated H3K9me was counted manually by ImageJ software (right). Scale bars, 10 μm. (d) Representative images of immunofluorescence staining of pH2AX and 53BP1 in J, DSJ, and RSJ (right). Quantitation of pH2AX and 53BP1 in J, DSJ, and RSJ (left). Scale bars, 10 μm. (f) Histogram of flow cytometry with PI staining to address the cell cycle of senescent J774A.1 macrophages showing the cell proportions in different phases of G1 and G2/S. (g) Top: scanning electron microscopy (SEM) images of J, DSJ, and RSJ with more phagocytic vacuoles than the controls. Scale bar, 200 μm. Bottom: transmission electron microscopy (TEM) images of J, DSJ, and RSJ showing the mitochondrial shrinkage with increased electron density (white arrow), endoplasmic reticulum expansion with degranulation (red arrow) and phagocytic vacuoles (blue arrow). Scale bar, 500 nm. (h) Representative fluorescent images of phagocytic clearance in R, DSR, and RSR. Scale bars, 100 μm. (i) Representative fluorescent images of phagocytic clearance in J, DSJ, and RSJ. Scale bars, 100 μm. (j, k) Representative images of wound healing assay for RAW264.7/J774A.1. Data were obtained at 24 and 48 h after scratch. Scale bars, 200 μm. (l, m) Representative image of senescent J774A.1/RAW264.7 cells under the inverted microscope and histogram indicating the average diameters of senescent macrophages (l: DSR, and RSR, m: R, J, DSJ, and RSJ). Scale bars, 50 μm (upper) and 20 μm (bottom). *n* = 3 in each group from independent biological replicates (d, f, k), *n* = 4 in each group from independent biological replicates (l, m), *n* = 6 in each group from independent biological replicates (c, e). Statistical significance was determined by one‐way ANOVA with Tukey's post hoc test; mean ± s.d. (c, d, e, f, k, l, m).
**Figure S3:** Additional characterization of D‐galactose, cisplatin or radiation induced senescent cells. (a) RAW264.7 cells were treated by D‐galactose (D‐galactose ‐induced senescent RAW264.7 cells, GSR) and stained for SA‐β‐Gal (left). Quantitation of percent SA‐β‐Gal^+^ cells (right). Scale bar, 100 μm. (b) Representative fluorescent images of H3K9me in R and GSR. Scale bar, 5 μm. (c) Immunoblots of senescence‐associated proteins, including p16, p21, p53, Bcl‐2, and Bcl‐XL in R, DSR, and RSR, respectively. (d, f) Immunoblots and quantitation protein expression of senescence‐associated proteins in J, DSJ, and RSJ, respectively. (e, g) Immunoblots and quantitation protein expression of senescence‐associated proteins in R and GSR. (h) Histogram of flow cytometry with PI staining to address the cell cycle of senescent macrophages showing the cell proportions in different phases of G1 and G2/S. (i) R and GSR were measured for cell proliferation by the CCK‐8 assay. (j) R, DSR, and RSR were measured for cell proliferation by the CCK‐8 assay. (k). J, DSJ, and RSJ were measured for cell proliferation by the CCK‐8 assay. (l) Relative expression of CD86, TNFa, iNOS, CD206, IL‐10 and arginase1 in R, DSR, RSR, J, DSJ, and RSJ. *n* = 3 in each group from independent biological replicates (f, g, h, i, j, k, l), *n* = 4 in each group from independent biological replicates (a, b). Statistical significance was determined by one‐way ANOVA with Tukey's post hoc test; mean ± s.d. (a, i, j, k, l); unpaired, two‐tailed t‐test; mean ± s.d. (b, f, g, h).
**Figure S4:** Senescent macrophages exhibit altered glutamine metabolism and elevated IL‐1β production. (a) Volcano plots showing the ‐log_10_ (*p* value) and log_2_ (Fold change) of metabolites in control (R) and senescent macrophages (DSR and RSR). The red points were metabolites with *p* < 0.05 and fold change > 1, and the blue points were metabolites with *p* < 0.05 and fold change < 1. (b) Bubble plots showing the top 20 pathways in the KEGG enrichment analysis results of differential metabolites between senescent macrophages (DSR and RSR) and the control (R). The color gradient from green to red indicates that the *p*‐value decreased. The size of the point was positively correlated with the number of metabolites clustered on the pathway. (c) Venn diagrams showing the same part of the pathway of the top 20 KEGG enrichment analysis results in both senescent macrophages (DSR and RSR). (d) Bar diagram showing the number of pathways involved by 28 metabolites in the same part of the pathway in the top 20 KEGG enrichment analysis of the two senescence macrophages. (e) Heat‐map showing changes in genes related to central carbon metabolism, alanine metabolism, aspartic acid metabolism, and glutamine metabolism in R, DSR and RSR (*n* = 3). (f) Glutamine consumption in control (J) and senescent macrophages (DSJ, RSJ). (g‐i) ATP, glucose consumption and lactate production in R, J, DSR, DSJ, RSR, or RSJ, respectively. *n* = 3 in each group from independent biological replicated. (j) Glutamate concentration in CM from J, DSJ, and RSJ, respectively. (k) Quantitative analysis results of glutamate content in the supernatant after using C‐IN‐1 to intervene in senescent macrophages (DSJ, RSJ). (l) Relative expression of GLS2 in DSJ and RSJ from J774A.1 cells. (m) Representative immunoblots showing GLS2 protein levels in DSR, DSJ, RSR and RSJ. Statistical significance was determined by one‐way ANOVA with Tukey's post hoc test; mean ± s.d. (f–m).
**Figure S5:** Transcriptomic and metabolic reprogramming of senescent macrophages reveals enhanced inflammatory signaling and altered energy metabolism. (a) PCA of gene expression in R, DSR, or RSR, respectively. Colored circles represent 95% confidence intervals, and colors correspond to different groups. (b‐c) Heat‐map showing the expression of top 100 significantly changed genes sequenced by the absolute value of log_2_ (Fold change). Venn diagrams showed the shared the significantly changed genes in DSR and RSR. (e) Volcano plot showing the −log_10_ (*p* value) and log_2_ (Fold change) of genes in R, DSR, or RSR, respectively. The red points were metabolites with *p* < 0.05 and Fold change > 2, and the blue points are metabolites with *p* < 0.05 and Fold change < 1. (d) Gene Set Enrichment Analysis (GSEA) gene sets network showing the results in DSR, RSR, and gene sets with the same function connected to each other. The color of the bubbles corresponded to the NES, and the size of the bubbles correlated with the number of genes within the gene base. (f) GSEA results of macrophages (DSR and RSR) in mitochondrial respiratory chain complex assembly, citric acid cycle TCA cycle, the citric acid TCA cycle and respiratory electron transport, pyruvate metabolism and citric acid TCA cycle and mitochondrial TCA cycle enzyme complex sets. (g) Representative immunofluorescence image for IL‐1β staining, nuclear staining (DAPI) and merge in senescent macrophages (DSJ and RSJ) after different concentrations of C‐IN‐1 stimulation. (h) Mean fluorescence intensity of IL‐1β staining in R, J, DSR, DSJ, RSR, or RSJ, respectively. (i) Representative immunoblots showing the levels of IL‐1β in control and senescent macrophages (DSR, RSR, DSJ and RSJ) after 20 μM C‐IN‐1 stimulation (left). Quantitative analysis of IL‐1β immunoblots in the protein level (right). (j) ELISA of IL‐1β levels in DSJ or RSJ treated with 20 μM C‐IN‐1. (k), IL‐1β expression analysis under different glutamine‐deprivation conditions in DSJ or RSJ. Scale bar, 100 μm (g). *n* = 3 in each group from independent biological replicates (h‐k). Statistical significance was determined by one‐way ANOVA with Tukey's post hoc test; mean ± s.d. (h–k).
**Figure S6:** Additional senescent macrophages promote oral cancer invasion in vitro. (a) OD values at 450 nm of SCC‐7 cell culture medium measured after 1 or 2 days of co‐culture with blank control, R, DSR or RSR following the addition of the CCK‐8 reagent. (b) Representative colony formation images and quantitation of SCC‐7 cells co‐cultured with senescent macrophages for 1 or 2 days. Compared with controls, senescent macrophages did not significantly affect colony numbers but reduced colony size. (c) Representative SA‐β‐gal staining image for SCC‐7 cells after 2 days of co‐culture with no significant change. Scale bar, 100 μm. (d) Representative images showing expression of Ki‐67 by immunofluorescence staining in SCC‐7 cells after 2 days of DMEM alone, co‐culture with macrophages (R, DSR or RSR). Nuclei were stained with DAPI. Scale bar, 100 μm. (e‐g) Flow cytometry showing senescent macrophages inhibit the proliferative ability of SCC‐7 cells after 2 days of co‐culture, compared to the blank and control groups (R or J). (h, k) Representative images and quantification of migration in Transwell assays with conditioned medium (CM) or co‐culture with R, DSR or RSR, respectively. SCC‐7 cells were treated by CM derived from R, DSR or RSR, respectively. Scale bar, 100 μm. (i, j) Representative images showing migration and invasion levels in Transwell assays with conditioned medium (CM) or co‐culture with R, DSR or RSR, respectively. SCC‐7 cells were treated by CM derived from J, DSJ, or RSJ respectively. Scale bar = 100 μm. *n* = 3 in each group from independent biological replicates and statistical significance was determined by one‐way ANOVA with Tukey's post hoc test (a, b, d, e, f, k).
**Figure S7:** Additional senescent macrophages secrete IL‐1β for promoting oral cancer invasion in vitro and in vivo. (a, b) Representative images showing wound healing levels for SCC‐7 cells conditioned medium (CM) or co‐culture with senescent macrophages. SCC‐7 cells were treated by CM derived from R, DSR or RSR, and J, DSJ, or RSJ respectively. Scale bar, 200 μm. (c) Wound closure rate (%) within the 18 h for SCC‐7 cells co‐cultured with a Transwell chamber or CM. (d) Representative images of immunofluorescence staining of E‐cadherin and Vimentin staining in SCC‐7 cells co‐cultured with a Transwell chamber of in alone, J, DSJ or RSJ, respectively. Scale bar, 50 μm. (e) Schematic of the *Cdkn2a* ^*−/−*^ (*p16* ^*−/−*^) genetic mouse model. To Establish of complete knockout C57BL/6J mice via CRISPR/Cas9 strategies (KO mice), exon1 was removed and identified by PCR. The genotypes were determined to be *Cdkn2a* ^*−/−*^ (homozygote), *Cdkn2a* ^*+/−*^ (heterozygote) and *Cdkn2a* ^*+/+*^ (wild type). (f) Representative image of *Cdkn2a* knockdown gene mouse identification. The numbers 1 were *Cdkn2a* ^*+/+*^ wild type mice, the numbers 2, 3, 4, 5, and 8 were heterozygous *Cdkn2a* ^*−/−*^ genetic mice, and the numbers 6, 7 were homozygous *Cdkn2a* ^*−/+*^ genetic mice. (g) Schematic of the experimental approach for establishing a transplanted tumor model in the tongue. (h‐i) Representative immunofluorescent images with co‐expression of E‐cadherin and Vimentin and tongue weight in BALB/c nude mice bearing tumors from SCC‐7 cells alone (*n* = 9) or with R (*n* = 9), DSR (*n* = 10), RSR (*n* = 7), respectively. Scale bar, 20 μm. (j) Representative immunofluorescent images with co‐expression of E‐cadherin and Vimentin and tongue weight in *p16* ^−/−^ C57BL/6J mice bearing tumors from SCC‐7 cells alone (*n* = 8) or with R (*n* = 8), DSR (*n* = 8), RSR (*n* = 8), respectively. Scale bar, 20 μm. (k) Relative mRNA expression of GM‐CSF, IL‐1α, and IL‐1β in the DSJ or RSJ from J774A.1 cells, respectively. (l) Representative immunofluorescence staining images of IL‐1α levels in the intracellular protein of R, DSR, and RSR, or J, DSJ, and RSJ. Scale bars, 50 μm. (m) Upregulated IL‐1β levels in the supernatants of DSJ and RSJ were determined by ELISA. (n) Immunoblots and representative immunofluorescence staining images of IL‐1β levels in the intracellular protein of J, DSJ, and RSJ. Scale bars, 50 μm. *n* = 3 in each group from independent biological replicates and statistical significance was determined by one‐way ANOVA with Tukey's post hoc test; mean ± s.d. (c, m, n).
**Figure S8:** Additional exogenous recombinant IL‐1β promotes oral cancer invasion and blocking IL‐1β attenuates this process and downstream signaling pathway. (a) Representative images and quantification of migration assays. Human derived cell lines including normal oral keratinocytes (NOK), dysplastic oral keratinocytes (DOK), tongue squamous cell carcinoma cells (CAL‐27) and OSCC cells (HSC‐3) were treated with exogenous IL‐1β. Scale bar, 100 μm. (b‐d) Representative images and quantification showing wound healing levels in NOK, DOK, CAL‐27 and HSC‐3 treated by recombinant IL‐1β. Scale bar, 100 μm. (e) Representative images and quantification of migration assays after treatment with DSR or RSR with an anti‐IL‐1β antibody. Scale bar, 100 μm. (f, h) Representative images and quantification of migration (f) and invasion (h) assays after treatment with DSJ or RSJ with an anti‐IL‐1β antibody. Scale bar, 100 μm. (g) Representative images of immunofluorescence staining of E‐cadherin and Vimentin in SCC‐7 co‐cultured indirectly with DSJ or RSJ treated with or without anti‐IL‐1β antibody, respectively. Scale bars, 100 μm. *n* = 3 in each group from independent biological replicates and statistical significance was determined by one‐way ANOVA with Tukey's post hoc test; mean ± s.d. (a, d, e, f, h).
**Figure S9:** Additional inhibition of IL‐1β reduces vimentin and increases E‐cadherin to suppress OSCC invasion in vivo. (a) Representative images of tumor sections from S‐RSR treated by anti‐IL‐1β antibody alone, C‐IN‐1 alone, and a combination with anti‐IL‐1β antibody and C‐IN‐1. (b) Schematic of the experimental approach for establishing a xenograft mouse model. First, SCC‐7 cells were injected subcutaneously with DSR or RSR for tumor formation. Secondly, an anti‐IL‐1β antibody suspended in 15% w/v PLGA‐PEG‐PLGA gel was injected around the tumor site one week later. Two weeks later, the tumor samples were harvested for IHC and mIHC staining procedures. (c) Representative images of tumors from nude mice implanted with SCC‐7 cells alone and co‐implanted with DSR or RSR treated with vehicle or anti‐IL‐1β antibody. (d) Representative images and quantification showing Transwell migration and invasion levels for SCC‐7 cells co‐cultured RSR in a Transwell chamber treated by anti‐IL‐1β antibody alone, C‐IN‐1 alone, and a combination with anti‐IL‐1β antibody and C‐IN‐1. (e) Tumor weight from nude mice co‐implanted with SCC‐7 cells and RSR. (f) Representative images and quantification of HIF‐1α and PKM2 in tumor sections from S‐RSR treated by anti‐IL‐1β antibody alone, C‐IN‐1 alone, a combination with anti‐IL1‐β antibody and C‐IN‐1 (*n* = 7 independent biological replicates for each group). (g) Tumor weight of nude mice from SCC‐7 cells alone and co‐implanted with DSR or RSR treated with vehicle or anti‐IL‐1β antibody (*n* = 6 in the comparisons except *n* = 4 in the group of SCC‐7 cells alone + vehicle). (h) Representative IHC images of E‐cadherin and vimentin, or IL‐1R1 and IL‐1R2 in nude mice bearing tumor from SCC‐7 cells alone, S‐DSR or S‐RSR treated with or without the anti‐IL‐1β antibody. Statistical significance was determined by one‐way ANOVA with Tukey's post hoc test; mean ± s.d. (d, e, f, g).

## Data Availability

Genomic datasets are available from the Gene Expression Omnibus database (GEO) under accession codes GSE234933, GSE173855, and GSE65858. The TCGA OSCC data are available at https://xena.ucsc.edu/. The datasets supporting the conclusions of this article are included within the article and its additional files.
