# Supplementary material for: Oxidative Stress Induced Senescent Macrophage‐Driven Squamous Cell Carcinoma Invasion via Glutamine Metabolic Reprogramming
Source: Aging Cell. 2026 Jun 23;25(7):e70592. doi: 10.1111/acel.70592 (PMC13288175; doi:10.1111/acel.70592)
Supplement: Supplementary file 1 — Figure S1: Additional differential infiltrations of senescent macrophages promote oral cancer progression. (a) Representative H&E staining and IHC images of CD68, p16, Ki‐67, p53, E‐cadherin and vimentin staining in human oral tissues. Scale bars, 100 μm for H&E, 50 μm for IHC. (b) Representative mIHC images showing the co‐expression of CD68 and p16 proteins in human oral normal mucosa, OLK, and OSCC patients. Scale bars, 25 μm (left) and 50 μm (right). (c) Co‐expression of CD68, p16 and Ki‐67 in mIHC staining images. Scale bars, 25 μm (upper), 50 μm (bottom). (d) Representative mIHC images showing the co‐expression of CD68, p16, and p53 proteins in human oral normal mucosa, OLK, and OSCC patients. Scale bars, 100 μm (upper), 50 μm (bottom). (e, f) Feature plot (c) and UMAP plot (d) of scRNA‐seq data showing all cells from OSCC tumors with metastasis were identified as 17 groups of cells. (g) GSEA enrichment plot of bulk RNA sequencing of senescence‐associated genes between non‐metastasis OSCC and OSCC with metastasis. Statistical significance was determined by permutation analysis in GOBP Cellular Senescence, FRIDMAN Senescence, and SEN MAYO sets. Figure S2: Additional characterization of cisplatin or radiation induced senescent cells. (a) Schematic of the in vitro experiments for establishing of drug‐induced senescent macrophages and radiation‐induced senescent macrophages. (b, d) J774A.1 cells (J) were treated by cisplatin (drug‐induced senescent J774A.1 cells, DSJ) or radiation (radiation‐induced senescent J774A.1 cells, RSJ) and stained for SA‐β‐Gal (b). Quantitation of percent SA‐β‐Gal+ cells (d). Scale bars, 100 μm. (c) Representative fluorescent images of H3K9me in J, DSJ, and RSJ. The accumulated H3K9me was counted manually by ImageJ software (right). Scale bars, 10 μm. (d) Representative images of immunofluorescence staining of pH2AX and 53BP1 in J, DSJ, and RSJ (right). Quantitation of pH2AX and 53BP1 in J, DSJ, and RSJ (left). Scale bars, 10 μm. (f) Hist [file ACEL-25-e70592-s001.docx]

**Oxidative stress induced senescent macrophage-driven squamous cell carcinoma invasion via glutamine metabolic reprogramming**

Shimeng Wang^1,2#^, Jingtian Mu^1#^, Wei Zhao^1^, Can Hu^1^, Xueke Shi^1^, Hongmei Zhou^1*^, Junjiang Liu^1,3*^, Fanglong Wu^1*^

**Author details**

^1^State Key Laboratory of Oral Diseases & National Center for Stomatology & National Clinical Research Center for Oral Diseases & Frontier Innovation Center for Dental Medicine Plus, West China Hospital of Stomatology, Sichuan University, Chengdu 610041, Sichuan, China.

^2^Department of Pathology and Laboratory Medicine, School of Medicine, University of California, Davis, Sacramento 95816, CA, USA.

^3^Department of Stomatology, Affiliated Hospital of North Sichuan Medical College, Nanchong, China.

^#^These authors contributed equally: Shimeng Wang and Jingtian Mu.

^*^These authors jointly supervised this work: Hongmei Zhou, Junjiang Liu and Fanglong Wu.

1. mail: zhouhm@scu.edu.cn; liujunjiang@nsmc.edu.cn and wufanglong@scu.edu.cn

**Supplementary Methods**

**Cell lines**

Murine macrophage cell line RAW264.7 was obtained from Shanghai ZQXZBIO. Murine macrophage cell line J774A.1 and OSCC cell line SCC-7 was obtained from BeNa Culture Collection (BNCC). Human normal oral keratinocyte (NOK) and human oral precancerous cell (DOK) were obtained from the State Key Laboratory of Oral Diseases, Sichuan University. Human OSCC cell line HSC-3 was obtained from JCRB Cell Bank. Human OSCC cell line CAL-27 was obtained from American Type Culture Collection (ATCC). Cells were cultured in Dulbecco's Modified Eagle Medium (DMEM, Gibco) supplemented with 10% fetal bovine serum (FBS, Bovogen, France) and 1% Penicillin-Streptomycin Solution (Gibco). During glutamine deprivation experiments, cells were cultured in DMEM without L-glutamine (Gibco).

**Establishment of senescent macrophages**

RAW264.7 and J774A.1 cells were cultured in 10 cm dishes until they reached 50% confluency. Cells were divided into groups for subsequent treatments. For radiation-induced senescence, cells were exposed to ionizing radiation at doses of 0 Gy, 4 Gy, and 10 Gy to stimulate macrophage senescence. For drug-induced senescence, cells were treated with complete culture medium containing cisplatin at concentrations of 0 μg/mL, 0.5 μg/mL, 1 μg/mL, and 2 μg/mL for 24 hours. After cisplatin or radiation treatment, cells were inoculated in a 6-well plate. After 1, 3, and 5 days of continued statics culture, cells were evaluated for senescence to determine the optimal stimulation concentration or dose.

**Conditioned medium preparation from macrophages**

5 × 10^6^ macrophages in 10 cm dishes were incubated in 10 mL of serum-free DMEM for 48 hours and then the cell supernatant were collected as conditioned medium (CM). To remove cells and cellular debris, the CM was centrifuged at 3,000 × g for 15 minutes and then filtered through a 0.22-μm filter (Merck Millipore). The CM would be stored at −80 °C.

**Senescence-associated β-galactosidase (SA-β-gal) staining**

Cells were cultured in 12-well plates (Corning). The CM was removed, and cells were washed three times with phosphate-buffered saline (PBS, Gibco) and then were fixed at room temperature with 2% formaldehyde solution (Biosharp, Cat# BL539A) for 15 minutes. The working solution was prepared according to the instructions of the SA-β-Gal staining kit (Beyotime, Cat# C0602) and incubated at 37 °C in a CO_2_-free and dark environment. Plates were sealed with parafilm to prevent evaporation of the staining solution. Overnight, the SA-β-Gal solution was removed and cells were washed with PBS and observed under a bright-field microscope. The blue or green cells were considered SA-β-Gal positive cells and then the percentage of SA-β-Gal positive cells was quantified based on staining.

**Immunofluorescence assay**

Cells were cultured in 12-well plates (Corning) or confocal laser scanning dishes (Biosharp) with 20 mm bottom diameter size and were fixed with 2% paraformaldehyde for 15 minutes. Permeabilization was performed using 0.2% Triton X-100 (BioFroxx, Cat# 1139ml500) for 10 minutes for cytoplasmic or nuclear proteins. Blocking was carried out with 5% BSA solution (BioFroxx, Cat# 424GR100) for one hour, followed by incubation with primary antibodies at 4°C overnight. The following primary antibodies and their dilutions were used: anti-H3K9me (Huabio, Cat# M1112-3, 1:250 dilution), anti-pH2AX (Abmart, Cat# T56572F, 1:200 dilution), anti-53BP1 (Abcam, Cat# ab36823, 1:200 dilution), anti-IL1α (Abclonal, Cat# A22766, 1:200 dilution), anti-IL-1β (Abclonal, Cat# A16288, 1:200 dilution), anti-E-cadherin (Abclonal, Cat# A22333, 1:200 dilution) and anti-Vimentin (Abclonal, Cat# A19607, 1:200 dilution). Secondary antibodies and their dilutions included iFlour^TM^ 488-conjugated anti-rabbit (Huabio, Cat# HA1121, 1:400), iFlour^TM^ 488-conjugated anti-rabbit (Huabio, Cat# HA1121, 1:400), iFlour^TM^ 594-conjugated anti-mouse (Huabio, Cat# HA1126, 1:400) iFlour^TM^ 594-conjugated anti-rabbit (Huabio, Cat# HA1122, 1:400). Finally, the nuclei were stained with DAPI soulution (BL105A, Biosharp) for visualization. The images of cells within the well plates were captured using an inverted fluorescence microscope (Olympus), while cells in confocal laser scanning dishes were imaged using an Olympus FV3000 confocal system (Olympus), enabling the detailed observation of multiple markers in the same section.

**Electron microscopy**

**Scanning electron microscopy (SEM).** Cells were cultured on glass coverslips in 6-well plates Cells were fixed with 2.5% glutaraldehyde solution (Solarbio, Cat# P1126) for 15 minutes. For graded dehydration, absolute ethanol was diluted with double-distilled water (ddH_2_O) into gradients of 30%, 50%, 70%, 90%, and 100%. Cells were dehydrated twice at each concentration for 15 minutes per step. After complete drying of the cell surface, samples were observed and imaged under a scanning electron microscope (Zeiss, Germany).

**Transmission electron microscopy (TEM).** Macrophages were cultured following the method described above. Logarithmic-phase cells were collected into 15 mL centrifuge tubes (Corning). Cells were centrifuged at 1,000 rpm for 5 minutes, and the supernatant was discarded. Along the tube wall, 0.5% glutaraldehyde solution was gently added, and cells were carefully re-suspended. The tubes were left to stand at 4 °C for 10 minutes. Cells were then centrifuged at 1,500 rpm for 15 minutes, and the supernatant was carefully removed. Subsequently, 2.5% glutaraldehyde fixative (pre-cooled) was carefully added along the tube wall. The samples were temporarily stored at 4°C. Fixed cell samples were prepared into ultrathin sections for observation of ultrastructural features and imaging using a transmission electron microscope (Thermo).

**Cell viability assays**

Cell viability was assessed using the Cell Counting Kit-8 (CCK-8) assay (APExBIO, Cat# K1018). Cells were collected, re-suspended, and counted, with 5,000 cells seeded per well in a 96-well plate. Subsequently, a 10% CCK-8 reagent solution diluted in complete culture medium was added to each well, and plates were incubated at 37°C for one hour. Absorbance at 450 nm was measured using a microplate reader (BioTek Instruments). After the measurement, the reagent was replaced with fresh complete culture medium, and cell viability was re-evaluated under identical conditions.

**Cell-cycle analysis**

Cells were stained using the Propidium Iodide (PI) staining kit (KeyGen BioTech, Cat# KGA512). They were collected by centrifugation and washed twice with the staining buffer prepared according to kit instructions. After washing, cells were centrifuged at 15,000 *g* for 5 minutes at 4 °C, and the supernatant was discarded. Cell pellets were fixed overnight at 4 °C with 70% ethanol. The following day, cells were centrifuged again at 15,000 *g* for 5 minutes, and the supernatant was removed, leaving the cell pellet. RNAse (Biomed, Cat# SH403) was added following the kit instructions, and cells were incubated at 37 °C for 30 minutes. Subsequently, PI reagent was added, and cells were incubated in the dark at room temperature for 30 minutes. PI staining results were analyzed using a flow cytometer, and cell cycle distribution patterns were quantified using ModFit software.

**RNA isolation and real-time PCR analysis**

Total RNA was isolated from cells using TRIzol reagent (JianShi BioTech, Cat# TR205) according to manufacturer’s instructions. The RNA was quantified using a UV spectrophotometer (Thermo Fisher Scientific). A total of 1 μg of RNA was reverse-transcribed into complementary DNA (cDNA) using the cDNA RT Kit (Servicebio, Cat# G3330-100). For quantitative real-time PCR (qRT-PCR), a 20 μL reaction system in 96-well PCR plate (Nest, Cat# 402712) per well was prepared, which included 2 × SYBR Green qPCR Master Mix (Servicebio, Cat# G3322-15), PCR primers, and cDNA samples, following proportions specified by the manufacturer (Roche). Amplification was performed using a real-time fluorescence quantitative PCR system. Data were analyzed using the ∆∆Ct method. The primer pairs used in this study are shown as following. IL-1α primers: forward, 5’-TTGGTTAAATGACCTGCAACA-3’ and reverse, 5’-GAGCGCTCACGAACAGTTG-3’; IL-1β primers: forward, 5’-AGTTGACGGACCCCAAAAG-3’ and reverse, 5’-AGCTGGATGCTCTCATCAGG-3’; TNF-α primers: forward, 5’-CACTCTGGGTACGTGGGTG-3’ and reverse, 5’-CACAGGTGATAATGAGGACAGC-3’; GM-CSF primers: forward, 5’-GGCCTTGGAAGCATGTAGAGG-3’ and reverse, 5’-GGAGAACTCGTTAGAGACGACTT-3’; GLS2 primers: forward, 5’-CGTCCGGTACTACCTCGGT-3’ and reverse, 5’-TGTCCCTCTGCAATAGTGTAGAA-3’; CD86 primers: forward, 5’-TGTTTCCGTGGAGACGCAAG-3’ and reverse, 5’-TTGAGCCTTTGTAAATGGGCA-3’; CD206 primers: forward, 5’-CTCTGTTCAGCTATTGGACGC-3’ and reverse, 5’-CGGAATTTCTGGGATTCAGCTTC-3’; Arginine1 primers: forward, 5’-CTCCAAGCCAAAGTCCTTAGAG-3’ and reverse, 5’-AGGAGCTGTCATTAGGGACATC-3’; IL-10 primers: forward, 5’-GCTCTTACTGACTGGCATGAG-3’ and reverse, 5’-CGCAGCTCTAGGAGCATGTG-3’; iNOS primers: forward, 5’-GTTCTCAGCCCAACAATACAAGA-3’ and reverse, 5’-GTGGACGGGTCGATGTCAC-3’; GAPDH primers: forward, 5’-CAGTGGCAAAGTGGAGATTGTTG-3’ and reverse, 5’-TCGCTCCTGGAAGATGGTGAT-3’.

**Protein extraction and immunoblotting**

Total protein was extracted from cells using a Total Protein Extraction Kit (SAB Biotech, Cat# PE001). The cells in 6-well plates (Corning) were washed twice with pre-cooled PBS. Cell lysis buffer was prepared according to the manufacturer's instructions, and cells were lysed on ice for 15-30 minutes. The lysate was collected into 1.5 mL EP tubes (AxyGen, Cat# MCT-150-C-S) and subjected to ultrasonic disruption on ice to ensure complete cell lysis. The lysate was then centrifuged at 15,000 *g* for 15 minutes at 4 °C, and the supernatant containing soluble proteins was collected for further analysis. Protein concentration was measured using the BCA method (Beyotime, Cat# P0010), following the kit instructions. Equal amounts of protein lysates were separated by sodium dodecyl sulfate-polyacrylamide gel electrophoresis (Bio-rad, SDS-PAGE) and transferred onto polyvinylidene difluoride (Amersham, PVDF) membranes. The membranes were blocked at room temperature with 5% BSA for one hour, followed by overnight incubation at 4 °C with various primary antibodies. The membranes were then incubated with the appropriate horseradish peroxidase (HRP)-conjugated secondary antibodies (Huabio, Cat# HA1013 and HA1024, 1:10,000 dilutions) and visualized using enhanced chemiluminescence (ECL) reagents (APExBIO, Cat# K1231). The following primary antibodies were incubated overnight: anti-p16 (Abclonal, Cat# A11651, 1:1,000 dilution), anti-p53 (Abclonal, Cat# A0263, 1:1,000 dilution), anti-p21 (ZENBIO, Cat# R25235, 1:1,000 dilution), anti-Bcl-2 (Huabio, Cat# ET1702-53, 1:1,000 dilution), anti-Bcl-XL (Huabio, Cat# ET1603-28, 1:1,000 dilution), anti-Bax (Abcam, Cat# ab32503, 1:1,000 dilution), anti-GAPDH (Abclonal, Cat# A19056, 1:10,000 dilution), anti-IL1β (Abclonal, Cat# A16288, 1:1,000 dilution), anti-β-actin (Huabio, Cat# EM21002, 1:1,000 dilution), anti-GLS2 (Abclonal, Cat# A16029, 1:1,000 dilution), anti-IL1R1 (Immunoway, Cat# YT5263, 1:1,000 dilution), anti-IL1R2 (Abclonal, Cat# A1899, 1:1,000 dilution), anti-p57 (Abclonal, Cat# A6843, 1:1,000 dilution), anti-p27 (Abclonal, Cat# A19095, 1:1,000 dilution), anti-p65 (Abmart, Cat# T55034, 1:5,000 dilution), and anti-pp65 (Abmart, Cat# TP56372, 1:1,000 dilution).

**Phagocytosis Assay**

Cells were seeded into appropriate culture plates and allowed to adhere. Once cells adhered, phagocytic activity was evaluated using the Cell Meter™ Fluorimetric Phagocytosis Assay Kit (AAT Bioquest, Cat# 21225) following the manufacturer’s protocol. The culture medium was removed, and cells were washed with PBS to eliminate any residual medium. Fluorescent latex particles were prepared in pre-warmed complete medium and added to cells. The plates were incubated at 37°C for 4 hours to facilitate phagocytosis. Afterward, CytoTrace™ Green working solution was added to each well, followed by incubation at 37 °C for an additional 30 minutes. Following the incubation, cells were thoroughly washed with PBS to remove any non-internalized particles. Phagocytosis was observed using a Texas Red filter (Ex/Em = 570/600 nm) for the fluorescent particles and a FITC filter (Ex/Em = 490/525 nm) for CytoTrace™ Green.

**Wound Healing Assay**

Cells were seeded into 6-well plates and cultured until they reached full confluency (100%). A sterile 200 μL pipette tip (KIRGEN, Cat# KG1212) was used to create a straight scratch (wound) across the cell monolayer. Detached cells were removed by gently washing with PBS to ensure clean wound edges. Fresh serum-free medium or medium containing stimulation was added to each well. The plates were incubated at 37 °C in a humidified atmosphere with 5% CO_2_. Images of the wound area were captured at 0 hours and at subsequent time intervals using an inverted microscope. The wound closure rate was quantified by measuring the wound area at each time point relative to the initial wound area (0 hour) using ImageJ software.

**Transwell Migration and Invasion Assays**

**Migration Assay.** Transwell chambers with 8 μm pore membranes and 6.5 mm bottom diameter (Corning) were utilized for the Transwell migration assay. The lower chamber was filled with complete medium containing 10% FBS to act as a chemoattractant. Cells were suspended in serum-free medium, and 200 μL of the cell suspension with or without stimulation was added to the upper chamber. The chambers were incubated at 37 °C in a 5% CO₂ atmosphere for suitable hours. Following incubation, cells that migrated to the lower side of the membrane were fixed with 4% paraformaldehyde for 15 minutes, stained with 0.1% crystal violet (Biosharp, Cat# BL802A) for 5-10 minutes, and washed thoroughly with PBS. Stained cells were visualized using an inverted microscope, and the number of migrated cells was quantified using ImageJ software.

**Invasion Assay.** The Transwell invasion assay followed a similar protocol to the migration assay, with the exception that the Transwell membrane was pre-coated with Matrigel (Corning, Cat# 356234, ) to simulate the extracellular matrix. After suitable hours of incubation, cells that had invaded through the Matrigel and adhered to the underside of the membrane were fixed, stained, imaged, and counted using the same procedure as described for the migration assay.

**Colony Formation Assay**

Colony formation assays were performed to evaluate the proliferation capacity of SCC-7 cells co-cultured with senescent macrophages. SCC-7 cells were co-cultured with either senescent macrophages or non-macrophages for 2 days. After 2 days of co-culture, SCC-7 cells were seeded into 6-well plates at a density of 500 cells per well for 5 days to allow colony formation. Colonies were fixed with methanol and stained with crystal violet. Number of colonies and area of colony formation were measured using ImageJ software. Colony number is defined as the number of colonies larger than 100 pixel², and colony area refers to the proportion of the well area occupied by the aforementioned colonies in a six-well plate. Statistical analysis was performed to compare number of colonies and colony area between the groups.

**Liquid-Phase Chip Assay**

Cell culture supernatants were collected by centrifuging at 15,000 g for 10 minutes at 4 °C to remove debris and then filtered through a 0.22-μm filter. The detection of cytokines and chemokines was performed by Chengdu Milian Biotechnology Co., Ltd. Briefly, MILLIPLEX _MAP_ Mouse High Sensitivity T Cell Panel (Millipore, Cat# MHSTCMAG-70K) was used to test target cytokines and chemokines including CXCL1, CXCL2, GM-CSF, IL-1α, IL-1β, IL-2, IL-4, IL-5, IL-6, IL-7, IL-10, IL-12, IL-13, IL-17A, CXCL5, TNF-α, IFNγ, and MCP-1. Target cytokine and chemokine concentrations were quantified using the MILLIPLEX® MAP liquid-phase protein microarray system according to the manufacturer’s instructions.

**Transcriptomic Analysis**

To detect the total RNA expression level of cells, we used an Illumina HiSeq X10 performed by Majorbio Bio-pharm Biotechnology Co., Ltd (Shanghai, China). Briefly, the medium was discarded, and cells were quickly washed three with cold PBS. Then, they were scraped, collected into 1.5 mL EP tubes, and centrifuged at 1,000 rpm for 5 minutes at 4°C. Non-senescent and senescent RAW264.7 cell pellets (1 × 10^7^) were collected with at least three biological replicates for each group. Then, total RNA of cells was extracted from cells or tissue samples using an RNA extraction kit according to the manufacturer's protocol. The quality and concentration of RNA were assessed using a NanoDrop spectrophotometer and Agilent5300 to ensure RQN (RQN > 6.5). RNA samples meeting the quality criteria were used for subsequent library preparation. RNA-seq libraries were constructed using a commercial kit. Briefly, poly(A)+ RNA was enriched from total RNA using oligo (dT) beads, followed by fragmentation and cDNA synthesis. Adapters were ligated to the fragmented cDNA, and the resulting library was amplified using PCR. Transcript abundance was quantified using featureCounts, and differential expression analysis was performed with DESeq2 by R software (version 4.1.0, USA). An adjusted *P* value < 0.001 and | fold change | > 2 were set as thresholds for determining differential expression. Functional enrichment analysis, including Gene Ontology (GO) and KEGG pathway analyses, was performed to interpret the biological significance of differentially expressed genes.

**Bioinformatics analysis**

GSE65858 and TCGA datasets were downloaded. The log2 FPKM data of IL-1R1 and IL-1R2 were extracted from these databases to compare the differences between the paracancerous normal tissue and OSCC tissues. Then, GSE173855 dataset was also downloaded, which includes 7 OSCC tissues without metastasis and 4 OSCC tissues with metastasis. Four predefined gene sets, including FRIDMAN Senescence, GOBP Cellular Senescence, Purcell, and SEN_MAYO, were used for Gene Set Enrichment Analysis (GSEA) analysis. Differential expression analysis was first conducted to calculate the log2 fold change for each gene to generate a ranked gene list between the two groups,. GSEA was conducted using the clusterProfiler package in R software, with the ranked gene list as input and the aforementioned senescence-related gene sets. Enrichment scores (ES) were calculated for each gene set, followed by normalization to obtain normalized enrichment scores (NES). Statistical significance was assessed via 1,000 permutations, and false discovery rate (FDR) values were calculated to account for multiple comparisons. Gene sets with an false discovery rate (FDR) < 0.25 were considered significantly enriched and normalized enrichment scores (NES) > 1 was considered as promotive role.

**Metabolomic Analysis**

To detect the total metabolites expression level of cells, we used an ACQUITY UPLC I-Class system (Waters Corporation, Milford, USA) coupled with VION IMS QTOF Mass spectrometer (Waters Corporation, Milford, USA) by Shanghai Lu-Ming Biotech Co., Ltd (Shanghai, China). Briefly, the medium was discarded, and cells were quickly washed three times with cold PBS. Ccells were scraped, collected into 1.5 mL EP tubes, and centrifuged at 1000 rpm for 5 minutes at 4°C. Cell pellets of non-senescent RAW264.7 cell and senescent RAW264.7 cells were collected at a density of 1 × 10^7^ cells per group with at least three independent replicates for each cell line. Then, cell pellets were rapidly quenched by brief contact with liquid nitrogen. Cell samples were dissolved in 1 mL of pre-chilled methanol-water solution (volume/volume = 4:1), followed by the addition of 200 μL of chloroform and thorough vortexing. Samples were ultrasonicated on ice, and 20 μL of a 0.06 mg/mL methanol solution of L-2-chlorophenylalanine was added. After another round of ultrasonication on ice, samples were left to stand at −40°C for 30 minutes, then centrifuged at 13,000 rpm for 10 minutes at 4°C. A total of 800 μL of the supernatant was transferred to LC-MS vials, dried, and reconstituted with 300 μL of methanol-water solution. Reconstituted samples were left at −40°C for 2 hours, centrifuged again at 13,000 rpm for 10 minutes at 4°C, and 150 μL of the supernatant was filtered through a 0.22-μm filter into LC injection vials. Quality control (QC) samples were prepared by pooling equal volumes of all samples and analyzed alongside test samples. Metabolites were detected using an ACQUITY UPLC I-Class Plus system coupled with the QE Plus high-resolution mass spectrometer. Metabolite identification was performed by matching m/z values and retention times with databases including HMDB, METLIN, and KEGG. Data analysis was conducted using the online platform: [https://www.metaboanalyst.ca/.](https://www.metaboanalyst.ca/) An adjusted *P* value < 0.05, | fold change | > 1, and variable important in projection (VIP) > 1 were set as thresholds for determining differential metabolites expression.

**Enzyme-Linked Immunosorbent Assay (ELISA)**

To detect the IL-1α, IL-1β, and glutamine secretion level of cell culture supernatants, we used the following ELISA kits: IL-1α (RUIXIN BIOTECH, Ca# RX203082M), IL-1β (RUIXIN BIOTECH, Ca# RX203063M), and glutamine (RUIXIN BIOTECH, Ca# RXJ202919M) ELISA kits. Briefly, cells were centrifuged at 15,000 g for 10 minutes and then filtered through a 0.22-μm filter to remove debris. Working solutions for each kit component were prepared according to the manufacturer’s instructions. Samples and standards were added to pre-coated wells, followed by the addition of enzyme-conjugated reagents. The plates were incubated at 37 °C for one hour. A substrate solution was added, and the plates were incubated at 37 °C for 15 minutes to allow color development. The reaction was terminated with stop solution, and absorbance was measured at 450 nm within 15 minutes. Concentrations of IL-1α, IL-1β, and glutamine in the samples were quantified based on a standard curve generated from known concentrations of standards.

**Lactate Detection**

To detect the lactate secretion level of cell culture supernatants, we used a commercial Lactate Assay Kit (Nanjing Jiancheng Biotech, Ca# A020-2-2). Cell culture supernatants were collected and added the working solution. The reaction was then terminated, and absorbance was measured at 530 nm using a microplate reader. Lactate concentrations in the samples were calculated by comparing the absorbance values to a standard curve.

**Glucose Measurement**

To detect the glucose secretion level of cell culture supernatants, we used a commercial Glucose Assay Kit (Nanjing Jiancheng Biotech, Ca# A154-1-1). Cell culture supernatants were collected, centrifuged at 15,000 g for 10 minutes and then filtered through a 0.22-μm filter to remove debris. Standards and samples were added to a 96-well plate, with each sample run in duplicate. A working solution containing glucose oxidase and a colorimetric reagent were prepared according to the manufacturer’s instructions. Working solution was added to the wells, gently mixed, and incubated at 37 °C in the dark for 30 minutes. Absorbance was measured at 570 nm using a microplate reader. Glucose concentrations in the samples were calculated based on a standard curve.

**ATP assay**

The intracellular ATP content was measured using an ATP assay kit (Solarbio, Cat# BC0300) according to the manufacturer's instructions for preparing the cell extraction solution and working solution. Non-senescent macrophages and senescent macrophages were collected into a centrifuge tube, centrifuged at 1,000 rpm to discard the supernatant, and the extraction solution was added at a ratio of 500 μL per 1 mL of cells (about a density of 1 × 10⁴ cells). Cells were sonicated for 1 minute (ice bath, power 200 W, 2 seconds on, 1 second off) and then centrifuged at 10,000 g at 4 °C for 10 minutes. The supernatant was transferred to a new tube, and 500 μL of chloroform was added and mixed thoroughly. The sample was centrifuged at 10,000 g at 4 °C for 3 minutes, and the supernatant was collected and kept on ice for measurement. Working solution was added to the samples and standards, mixed thoroughly, and the absorbance at 340 nm was measured for 10 seconds (A1). The samples were then placed in a 37°C water bath for 3 minutes, after which the absorbance at 340 nm was measured again at 3 minutes and 10 seconds (A2). The ΔA measurement was calculated as follows: ΔA (sample) = A2 (sample) - A1 (sample); ΔA (standard) = A2 (standard) - A1 (standard). The final intracellular ATP content was calculated.

**Hydrogel preparation and drug loading**

PLGA-PEG-PLGA thermosensitive copolymer was used to prepare the injectable hydrogel. Briefly, the copolymer was accurately weighed and dissolved in deionized water under continuous magnetic stirring at 4 ℃ until a homogeneous and transparent solution was obtained. The prepared hydrogel precursor solution was stored at 4 ℃ prior to use. For in vivo administration, anti-IL-1β antibody or C-IN-1 was incorporated into the hydrogel by gentle mixing at low temperature to ensure uniform distribution. The resulting formulation remained in a sol state at low temperature and underwent a sol-gel transition at physiological temperature after injection, enabling localized and sustained delivery of the loaded agents.

**Mouse model**

Six- to eight-week-old male BALB/c nude mice (Beijing Vital River Laboratory Animal Technology Co., Ltd., China) were used as tumour recipients. Cdkn2a(*p16)^KO^* genetic C57BL/6J mice were established on the Jiangsu Wukong Biotechnology Co., Ltd, using CRISPR/Cas9 technology. And Six- to eight-week-old male *Cdkn2a(p16)^-/-^* genetic C57BL/6J mice were used as tumour recipients. All mice were maintained under pathogen-free conditions with a 12-hour light/dark cycle (6:00 AM to 6:00 PM), a temperature of 20 °C, and 50% humidity.

Animals were assigned to experimental groups using simple randomization. For establishing a model of OSCC in situ of tongue, anesthetized male athymic nude mice (BALB/c) and *p16^-/-^* genetic C57BL/6J mice received 1 × 10^4^ non-senescent or senescent macrophages and 1 × 10^4^ SCC-7 cells and subcutaneously transplanted into tongue with 50% Matrigel (Corning) in the following groups: SCC-7 cells alone, SCC-7 cells and non-senescent macrophages, SCC-7 cells and cisplatin-induced senescent macrophages (DSR), and SCC-7 cells and radiation-induced senescent macrophages (RSR). SCC-7 cells were used alone as the control group in this study. All viable cells were confirmed and quantified using an automatic Cell Counter (Countess3, Invitrogen) with Trypan blue staining. Two weeks’ post-implantation, mouse body weights were monitored. Tongue and tumour tissue samples were collected when mice experienced a 20% reduction in initial body weight, at which point euthanasia was performed. Responses were then scored of IHC or mIHC staining by an experimenter blinded to injection condition and experimental cohort.

To evaluate the effect of IL-1β inhibitor (anti-IL-1β antibody) and GLS-2 inhibitor (C-IN-1) treatment alone and in combination on liquid-phase OSCC in senescent macrophages, anesthetized male athymic nude mice (BALB/c) received 1 × 10^5^ SCC-7 cells and 1 × 10^5^ RSR and subcutaneously transplanted into the right flank with 50% Matrigel (Corning) in the following groups: SCC-7 cells + RSR + vehicle, SCC-7 cells + RSR + anti-IL-1β antibody, SCC-7 cells + RSR + C-IN-1, and SCC-7 cells + RSR + anti-IL-1β antibody + C-IN-1. Anti-IL-1β antibody (100 μg/week/mouse) and C-IN-1 (14 mg/week/mouse) treatments were administered after the tumour growth has stabilized. Treatment began after tumour formation, and euthanasia was conducted if body weight dropped by 20% of the initial weight or if the tumour exceeded ethically permissible sizes. Tumour tissues were collected one week after treatment. At the end of the study, tumours were weighed and measured at autopsy, ensuring they did not exceed maximum allowable sizes. Tumour volume was calculated using the formula:  1/2 (length × width^2^). The endpoint of the experiment was reached when any of the tumors exceeded the volume limit set by ethical guidelines.

To evaluate the effect of anti-IL-1β antibody on liquid-phase OSCC in senescent macrophages, anesthetized male athymic nude mice (BALB/c) received 1 × 10^5^ SCC-7 cells and 1 × 10^5^ macrophages, and subcutaneously transplanted into the right flank with 50% Matrigel (Corning) in the following test groups: SCC-7 cells + vehicle, SCC-7 cells + anti-IL-1β antibody, SCC-7 cells + DSR + vehicle, SCC-7 cells + DSR + anti-IL-1β antibody, SCC-7 cells + RSR + vehicle, and SCC-7 cells + RSR + anti-IL-1β antibody. Anti-IL-1β antibody (100 μg/week/mouse) treatments were administered after the tumour growth has stabilized. The following process is similar to above.

**Agarose Gel Electrophoresis**

To detect the genotype of *p16^KO^* genetic C57BL/6J mice, we used an extract mouse DNA using the One Step Mouse Genotyping Kit (Vazyme, Ca# PD101-01) for agarose gel electrophoresis. Prepare a 1% agarose gel according to the size of the DNA fragments to be analyzed. Dissolve agarose (Invitrogen, Cat# 75510019) in 1× TAE buffer (Biosharp, Cat# BL533A) by heating until completely melted. When the solution cools to approximately 50°C, add TS-GelRed nucleic acid stain (Tsingke Biotech, Cat# TSJ003,) and mix gently. Pour the gel into a casting tray with a comb to form wells, and allow it to solidify. Load the prepared DNA samples and a DNA ladder (Vazyme, Cat# MD101-01, ) carefully into the wells. Place the gel in an electrophoresis tank filled with 1× TAE buffer, ensuring the gel is fully submerged. Run the gel at a voltage of 120 V until the dye front has migrated a sufficient distance. Use a UV light gel documentation system (Bio-rad) to visualize the DNA bands. Compare positions of the sample bands with the DNA ladder to estimate sizes of DNA fragments. The primer sequences include followings: F1: 5’-GTACTGGTAACTCTGCCCAAAGC-3’, R1: 5’-CTTCCCCGTTAAAACTATCTACCCA-3’; F2: 5’-CTGGCCCTAGAAATGTCTGTAACTA-3’. Primer sequences of F1/R1 could produce a product of 362 bp and F2/R1 could produce a product of 459 bp. A mouse with only a 362 bp product was identified as *Cdkn2a^-/-^* (homozygote) genetic C57BL/6J mice. Similarly, a mouse with only a 459 bp product was identified as *Cdkn2a^+/+^* (wild type) C57BL/6J mice*.*

**Histology and immunostaining**

Paraffin-embedded tissues were cut into 4-μm thick sections and used for hematoxylin and eosin (H&E) staining or immunostaining procedures. H&E staining kit (Biosharp, Cat# BL700B) was performed for histological examination, following a previously established protocol. For immunohistochemistry (IHC), a Diaminobenzidine (DAB) detection kit (Elabscience, Cat# E-IR-R217,) was used. Sections were incubated overnight at 4 °C with the following primary antibodies.: anti-p16 (ABclonal Cat# A11651, 1:100 dilution), anti-CD68 (CST Cat# 97778T, 1:100 dilution), anti-Ki67 (Abclonal Cat# A23722, 1:100 dilution), anti-p53 (ABclonal Cat# A0263, 1:100 dilution, Abclonal), anti-E-cadherin (Abclonal Cat# A22333, 1:200 dilution), anti-Vimentin (ABclonal Cat# A19607, 1:200 dilution), anti-GLS2 (ABclonal Cat# A16029, 1:100 dilution, Abclonal), anti-IL1R1 (Immunoway, Cat# YT5263, 1:100 dilution), anti-IL1R2 (ABclonal Cat# A1899, 1:100 dilution), anti-HIF-1α (ABclonal Cat# A11945, 1:100 dilution), and anti-PKM2 (ABclonal Cat# A20991, 1:100 dilution). After incubation with primary antibodies, the sections underwent standard immunohistochemical processing. H&E and IHC-stained slides were examined using an Olympus SLIDEVIEW VS200 microscope.

Seven-color multiplex immunofluorescence (mIF) was carried out with a mouse—rabbit six-marker, seven-color multiplex immunofluorescence detection kit(Biodragon, Cat# BDAA0204). The fluorescent dyes included in this kit were D-488 (for CD68, 1:200 dilution), D-594 (for p53, 1:100 dilution), D-647 (for Ki67, 1:200 dilution), D-435 (for Vimentin, 1:200 dilution), D-525 (forP16 ^INK4A^ , 1:100 dilution), and D-750 (for E-cadherin, 1:100 dilution). Nuclear staining was performed using DAPI, which marked nuclei for clearer visualization. The slides stained with mIHC were imaged using and 3Dhistech (model no. 3D-histech) digital pathology scanner.

Multiplex immunohistochemistry (mIHC) was performed by a multicolor detection kit (Absin, Cat# abs50012). The kit employed TSA 520, TSA 570, and TSA 650 fluorescent channels, which allowed the visualization of different proteins simultaneously. Details of the targeted proteins detected via mIHC are provided in the figure legends. Nuclear staining was performed using DAPI, which marked nuclei for clearer visualization. The slides stained with mIHC were imaged using an Olympus FV3000 confocal system, enabling the detailed observation of multiple markers in the same section.

**Single-cell transcriptome analysis**

The scRNA-seq dataset GSE234933 was downloaded and included 8 OSCC tissues with metastasis. Quality control was conducted using the Seurat package (v4.3.0.1) in R (version 4.1.0, USA). Number of genes detected in each cell (nFeature_RNA), total number of molecules detected within a cell (nCount_RNA), percentage of mitochondrial transcripts (mt_percent), percentage of transcripts of red blood cells (HB_percent) were computed using the functions of the Seurat package. Quality control was performed as follows: (1) nFeature_RNA > 200 and nFeature_RNA < 4,000, (2) mt_percent < 10, (3) HB_percent < 3, (4) nCount_RNA > 1,000 and nCount_RNA < 30,000. Cells with doublet scores exceeding the simulated threshold determined by Scrublet package were considered potential doublets and excluded from the dataset. In total, 15,913 high-quality single cells passed quality control and were used for further analysis. Highly variable genes were identified with the function FindVariableGenes in Seurat following default parameters. These variable genes were then selected for downstream dimensionality reduction and clustering. PCA was performed on this scaled dataset. Cells were clustered based on the PCA scores of the first 30 principal components. The clustering of the data was performed based on the Seurat function FindClusters and cells were clustered into 17 groups. The sub-populations were identified according to marker genes (epithelial cells: EPCAM, KRT14, and KRT19; endothelial cells: PECAM1, VWF, and ENG; Fibroblasts: FAP, PDPN, and COL1A2; CD4^+^ T cells: CD3D, CD3E, IL7R, TCF7, PBXIP1, and FOXP3; CD8^+^ T cells: CD3D, CD3E, CD8A, and CD8B; NK cells: KLRD1 and KLRC1; B cells: CD19, MS4A1, and CD79A; Plasma cells: IGHG4 and SDC1; Monocytes: FCN1, S100A9, and S100A8; Macrophages: CD68, FCGR1A, and CD163; Dendritic cells: CD83, AOC1, and CD80; Plasmacytoid dendritic cells: IL3RA, TCF4, and RUNX2; Mast cell: TPSAB1 and TPSB2). After cell annotation, immune cells were clustered into 13 groups. Senescent cells were identified using four senescence-related gene sets: FRIDMAN Senescence, GOBP Cellular Senescence, Purcell, and SEN_MAYO. The above gene sets were obtained from the GSEA database (www.gsea-msigdb.org/gsea/index.jsp) and a study by Purcell et al (PMID: 25483067). Each cell was scored for senescence using the single-sample Gene Set Enrichment Analysis (ssGSEA) method implemented in the GSVA R package. Input data consisted of a log-normalized gene expression matrix and the gene sets in gmt format. Cells were ranked based on their ssGSEA scores, and the top 10% of cells with the highest scores were designated as senescent. Within the senescent cell subset, we calculated the proportion of each cell type. A stacked bar plot was generated to visualize the distribution of cell types among the senescent cells, providing insight into the contribution of different cell populations to the senescent phenotype.

**Supplementary Figures**


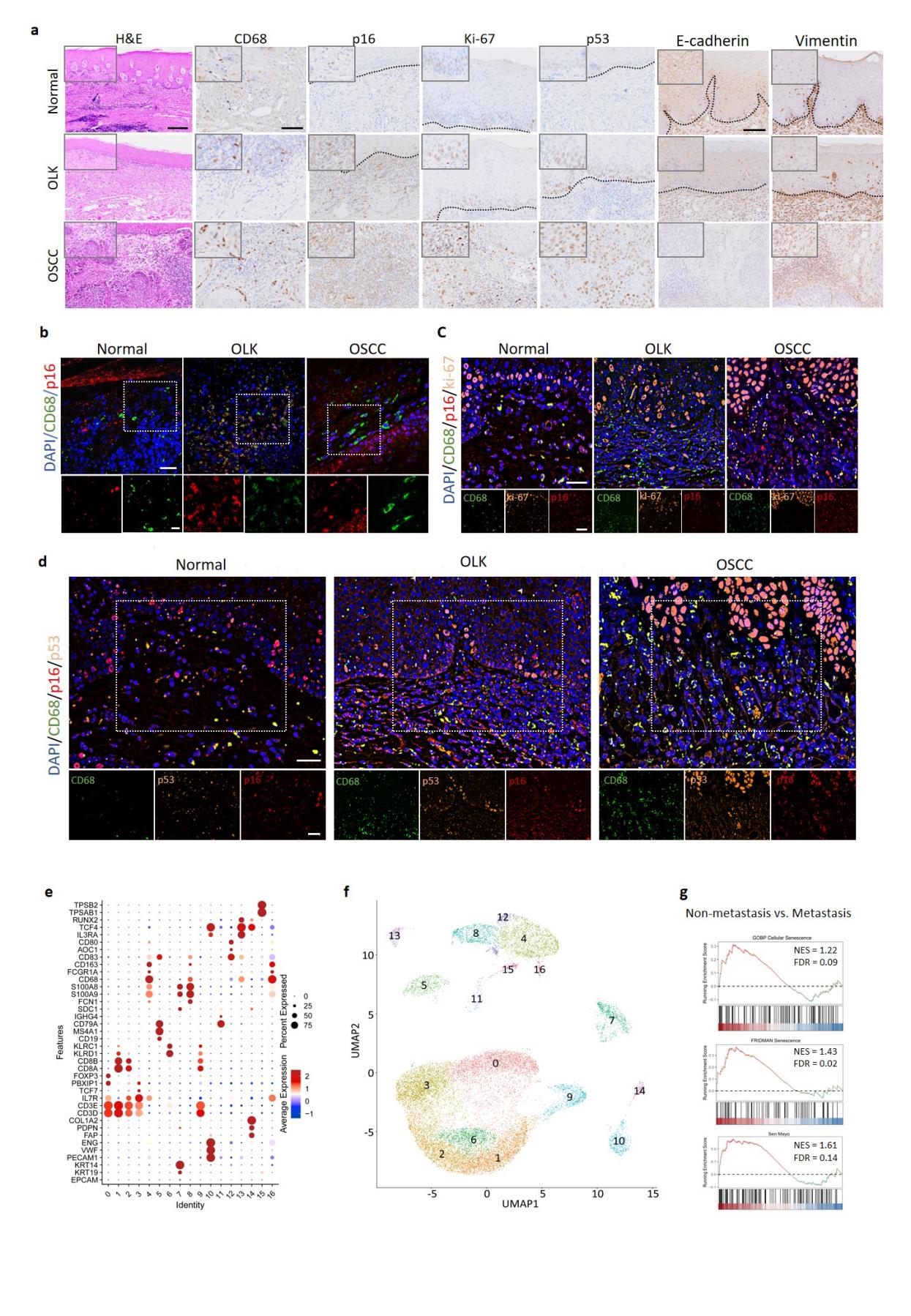


**Fig. S1 | Additional differential infiltrations of senescent macrophages promote oral cancer progression.** **a**. Representative H&E staining and IHC images of CD68, p16, Ki-67, p53, E-cadherin and vimentin staining in human oral tissues. Scale bars, 100 µm for H&E, 50 µm for IHC. b. Representative mIHC images showing the co-expression of CD68 and p16 proteins in human oral normal mucosa, OLK, and OSCC patients. Scale bars, 25 µm (left) and 50 µm (right). **c.** Co-expression of CD68, p16 and Ki-67 in mIHC staining images. Scale bars, 25 µm (upper), 50 µm (bottom). **d.** Representative mIHC images showing the co-expression of CD68, p16, and p53 proteins in human oral normal mucosa, OLK, and OSCC patients. Scale bars, 100 µm (upper), 50 µm (bottom). **e-f.** Feature plot **(c)** and UMAP plot **(d)** of scRNA-seq data showing all cells from OSCC tumours with metastasis were identified as 17 groups of cells. **g.** GSEA enrichment plot of bulk RNA sequencing of senescence-associated genes between non-metastasis OSCC and OSCC with metastasis. Statistical significance was determined by permutation analysis in GOBP Cellular Senescence, FRIDMAN Senescence, and SEN MAYO sets.

**
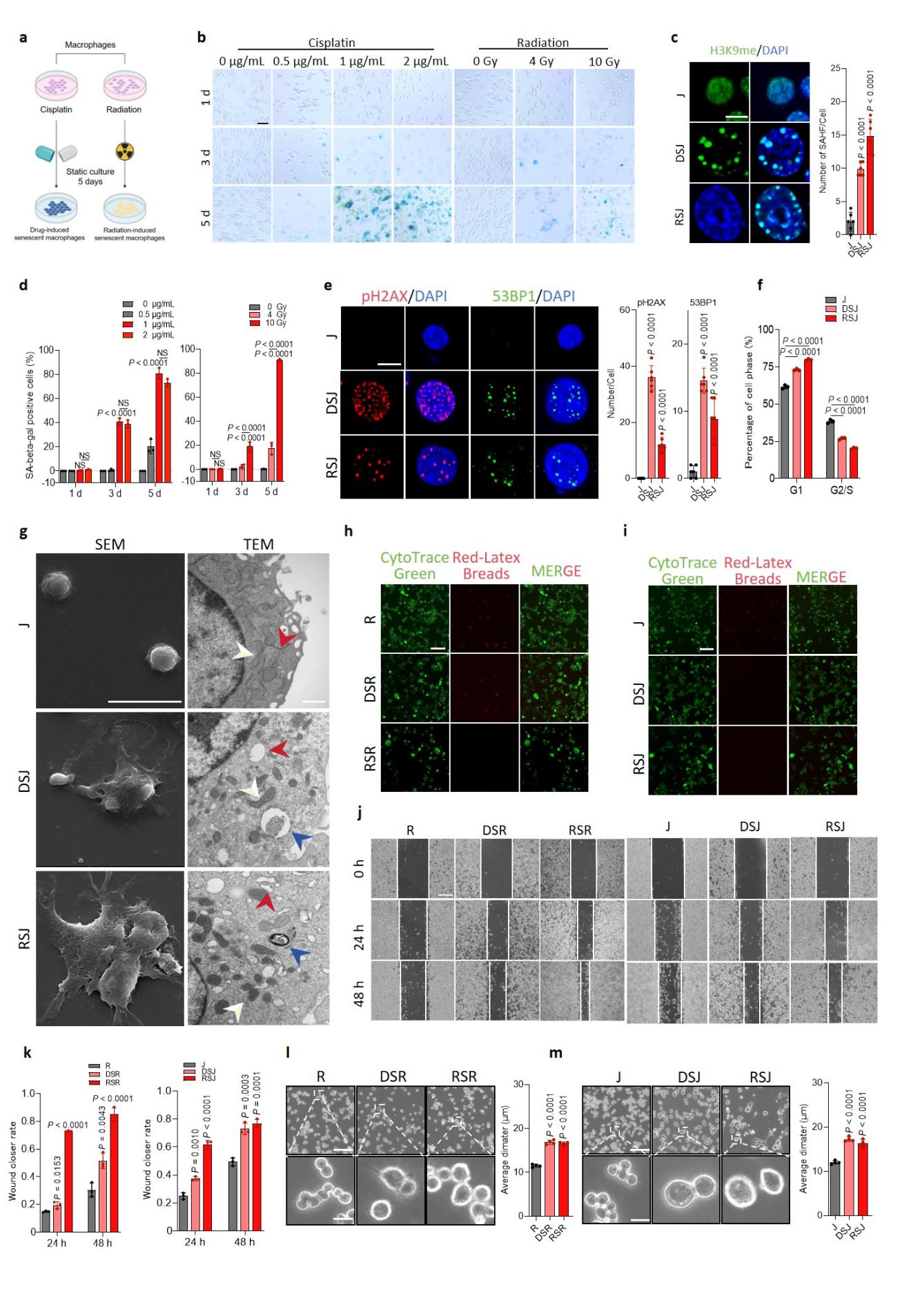
**

**Fig. S2 | Additional characterization of cisplatin or radiation induced senescent cells.** **a.** Schematic of the in vitro experiments for establishing of drug-induced senescent macrophages and radiation-induced senescent macrophages. **b, d**. J774A.1 cells (J) were treated by cisplatin (drug-induced senescent J774A.1 cells, DSJ) or radiation (radiation-induced senescent J774A.1 cells, RSJ) and stained for SA-β-Gal (**b**). Quantitation of percent SA-β-Gal^+^ cells (**d**). Scale bars, 100 µm. **c**. Representative fluorescent images of H3K9me in J, DSJ, and RSJ. The accumulated H3K9me was counted manually by ImageJ software (right). Scale bars, 10 µm. **d**. Representative images of immunofluorescence staining of pH2AX and 53BP1 in J, DSJ, and RSJ (**right**). Quantitation of pH2AX and 53BP1 in J, DSJ, and RSJ (**left**). Scale bars, 10 µm. **f.** Histogram of flow cytometry with PI staining to address the cell cycle of senescent J774A.1 macrophages showing the cell proportions in different phases of G1 and G2/S. **g**. Top: scanning electron microscopy (SEM) images of J, DSJ, and RSJ with more phagocytic vacuoles than the controls. Scale bar, 200 µm. Bottom: transmission electron microscopy (TEM) images of J, DSJ, and RSJ showing the mitochondrial shrinkage with increased electron density (white arrow), endoplasmic reticulum expansion with degranulation (red arrow) and phagocytic vacuoles (blue arrow). Scale bar, 500 nm. **h**. Representative fluorescent images of phagocytic clearance in R, DSR, and RSR. Scale bars, 100 µm. **i**. Representative fluorescent images of phagocytic clearance in J, DSJ, and RSJ. Scale bars, 100 µm. **j-k.** Representative images of wound healing assay for RAW264.7/J774A.1 . Data were obtained at 24 and 48 hours after scratch. Scale bars, 200 µm. **l-m**. Representative image of senescent J774A.1/ RAW264.7 cells under the inverted microscope and histogram indicating the average diameters of senescent macrophages (**l**: DSR, and RSR, **m**: R,J, DSJ, and RSJ). Scale bars, 50 µm (upper) and 20 µm (bottom). *n* = 3 in each group from independent biological replicates (**d, f, k**), *n* = 4 in each group from independent biological replicates (**l, m**), *n* = 6 in each group from independent biological replicates (**c, e**). Statistical significance was determined by one-way ANOVA with Tukey’s post-hoc test; mean ± s.d. (**c, d, e, f, k, l, m**).

**
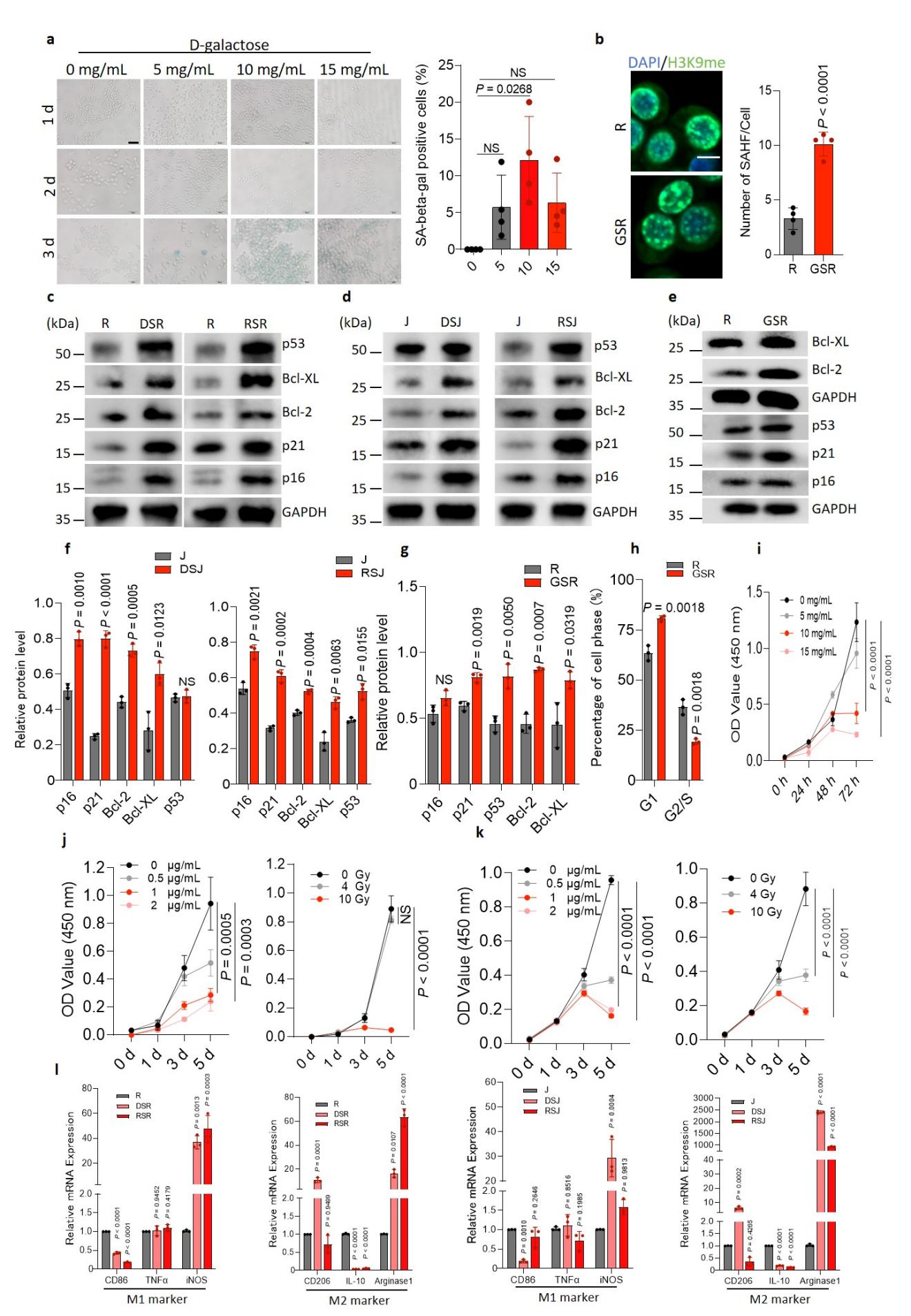
**

**Fig. S3 | Additional characterization of D-galactose,** **cisplatin or radiation induced senescent cells. a**. RAW264.7 cells were treated by D-galactose (D-galactose -induced senescent RAW264.7 cells, GSR) and stained for SA-β-Gal (left). Quantitation of percent SA-β-Gal^+^ cells (right). Scale bar, 100 µm. **b**. Representative fluorescent images of H3K9me in R and GSR. Scale bar, 5 µm. **c.** Immunoblots of senescence-associated proteins, including p16, p21, p53, Bcl-2, and Bcl-XL in R, DSR, and RSR, respectively. **d, f.** Immunoblots and quantitation protein expression of senescence-associated proteins in J, DSJ, and RSJ, respectively. **e, g.** Immunoblots and quantitation protein expression of senescence-associated proteins in in R and GSR. **h.** Histogram of flow cytometry with PI staining to address the cell cycle of senescent macrophages showing the cell proportions in different phases of G1 and G2/S. **i.** R and GSR were measured for cell proliferation by the CCK-8 assay. **j.** R, DSR, and RSR were measured for cell proliferation by the CCK-8 assay. **k.** J, DSJ, and RSJ were measured for cell proliferation by the CCK-8 assay. **l.** Relative expression of CD86, TNFa, iNOS, CD206, IL-10 and arginase1 in R, DSR, RSR, J, DSJ, and RSJ. n = 3 in each group from independent biological replicates (**f, g, h, i, j, k, l**), n = 4 in each group from independent biological replicates (**a, b**). Statistical significance was determined by one-way ANOVA with Tukey’s post-hoc test; mean ± s.d. (**a, i, j, k, l**); unpaired, two-tailed t-test; mean ± s.d. (**b, f, g, h**).


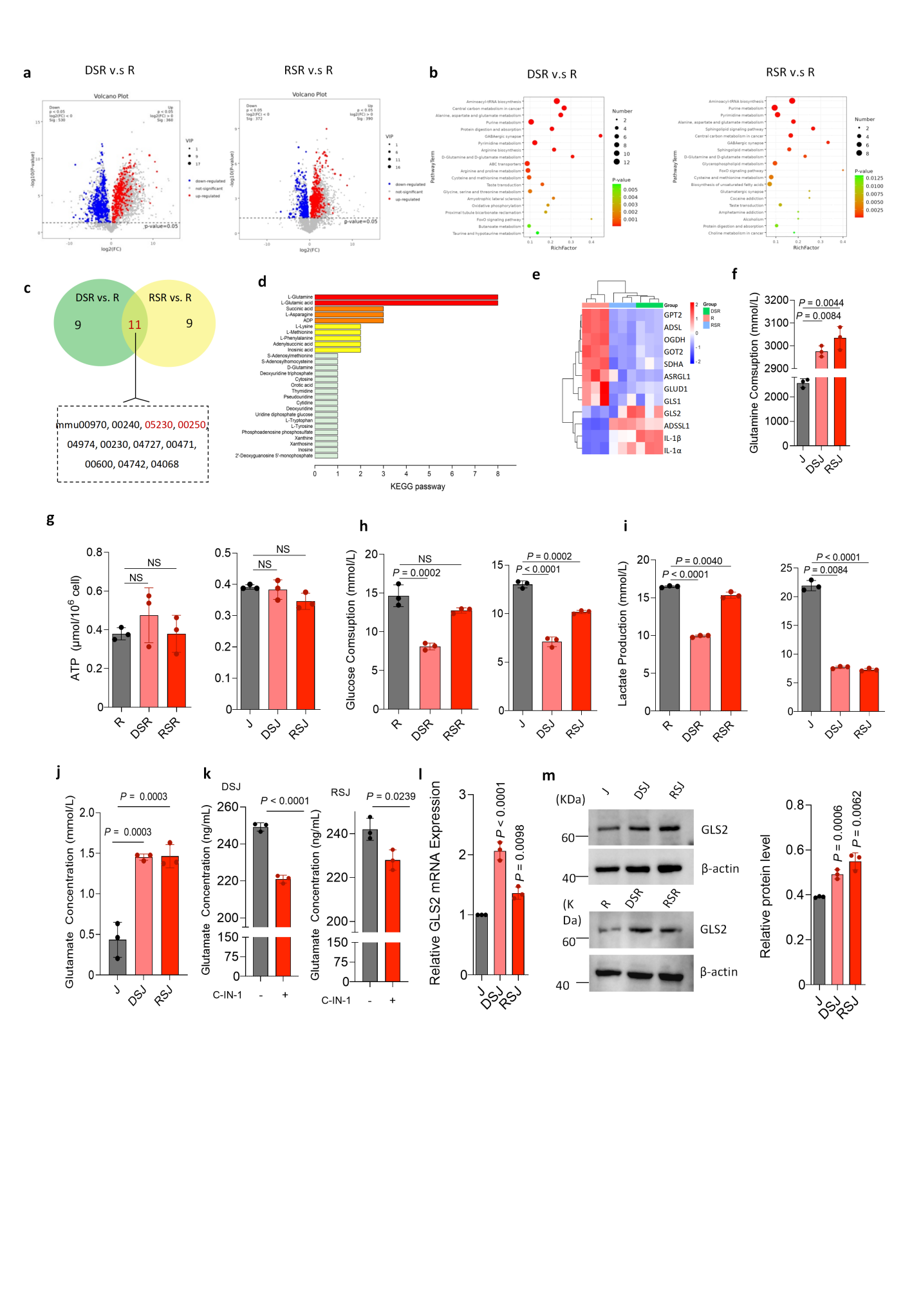


**Fig. S4 | Senescent macrophages exhibit altered glutamine metabolism and elevated IL-1β production. a**. Volcano plots showing the -log_10_ (*P* value) and log_2_ (Fold change) of metabolites in control (R) and senescent macrophages (DSR and RSR). The red points were metabolites with *P* < 0.05 and fold change > 1, and the blue points were metabolites with *P* < 0.05 and fold change < 1. **b**. Bubble plots showing the top 20 pathways in the KEGG enrichment analysis results of differential metabolites between senescent macrophages (DSR and RSR) and the control (R). The color gradient from green to red indicates that the *P*-value decreased. The size of the point was positively correlated with the number of metabolites clustered on the pathway. **c**. Venn diagrams showing the same part of the pathway of the top 20 KEGG enrichment analysis results in both senescent macrophages (DSR and RSR). **d**. Bar diagram showing the number of pathways involved by 28 metabolites in the same part of the pathway in the top 20 KEGG enrichment analysis of the two senescence macrophages. **e.** Heat-map showing changes in genes related to central carbon metabolism, alanine metabolism, aspartic acid metabolism, and glutamine metabolism in R, DSR and RSR (n = 3). **f.** Glutamine consumption in control (J) and senescent macrophages (DSJ, RSJ). **g-i.** ATP, glucose consumption and lactate production in R, J, DSR, DSJ, RSR, or RSJ, respectively. n = 3 in each group from independent biological replicated. **j**. Glutamate concentration in CM from J, DSJ, and RSJ, respectively. **k.** Quantitative analysis results of glutamate content in the supernatant after using C-IN-1 to intervene in senescent macrophages (DSJ, RSJ). **l**. Relative expression of GLS2 in DSJ and RSJ from J774A.1 cells. **m.** Representative immunoblots showing GLS2 protein levels in DSR, DSJ, RSR and RSJ. Statistical significance was determined by one-way ANOVA with Tukey’s post-hoc test; mean ± s.d. (**f-m**).

**
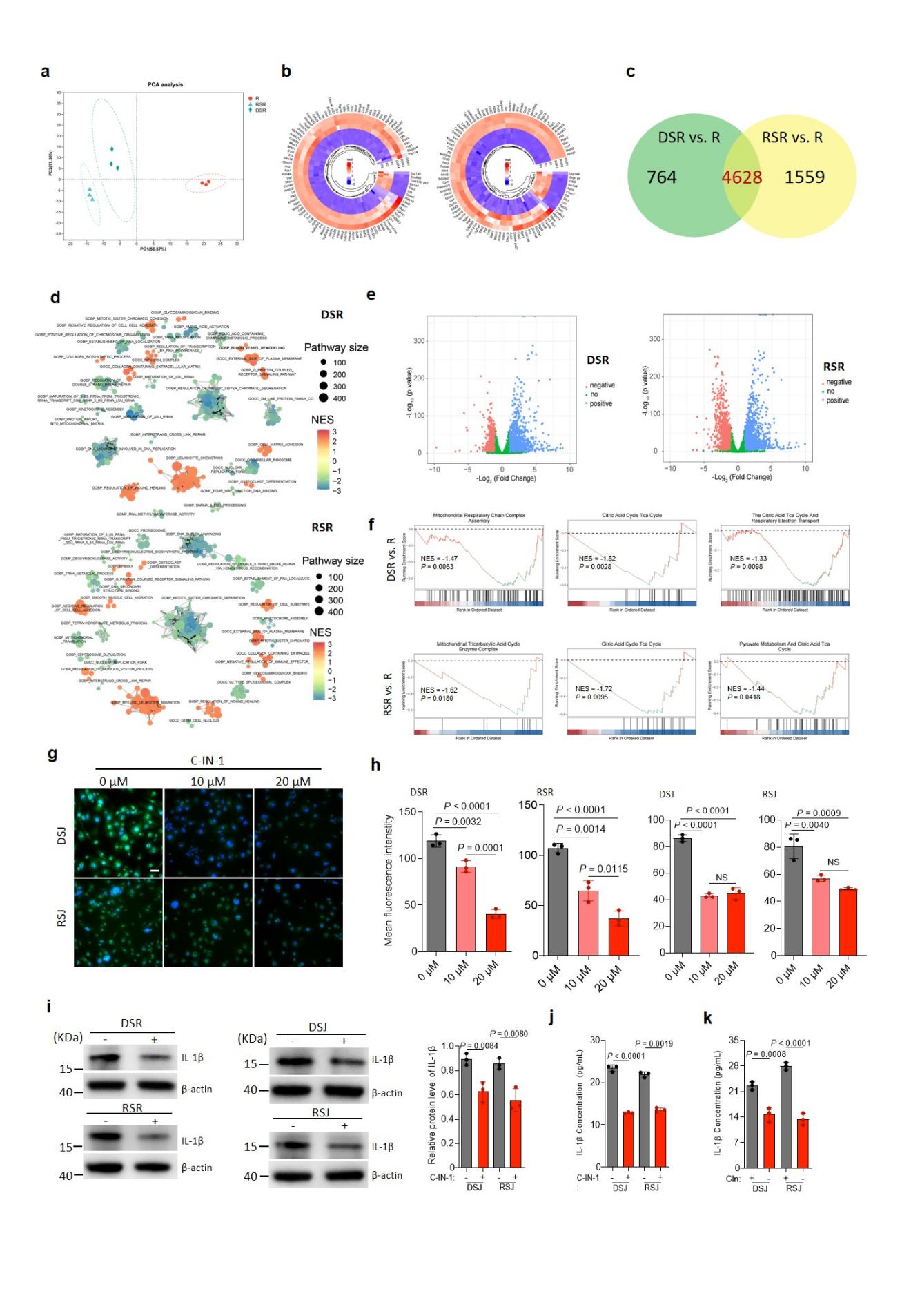
**

**Fig. S5 | Transcriptomic and metabolic reprogramming of senescent macrophages reveals enhanced inflammatory signaling and altered energy metabolism. a**. PCA of gene expression in R, DSR, or RSR, respectively. Colored circles represent 95% confidence intervals, and colors correspond to different groups. **b-c**. Heat-map showing the expression of top 100 significantly changed genes sequenced by the absolute value of log_2_ (Fold change). Venn diagrams showed the shared the significantly changed genes in DSR and RSR. **e**. Volcano plot showing the -log_10_ (*P* value) and log_2_ (Fold change) of genes in R, DSR, or RSR, respectively. The red points were metabolites with *P* < 0.05 and Fold change > 2, and the blue points are metabolites with *P* < 0.05 and Fold change < 1. **d**. Gene Set Enrichment Analysis (GSEA) gene sets network showing the results in DSR, RSR, and gene sets with the same function connected to each other. The color of the bubbles corresponded to the NES, and the size of the bubbles correlated with the number of genes within the gene base. **f**. GSEA results of macrophages (DSR and RSR) in mitochondrial respiratory chain complex assembly, citric acid cycle TCA cycle, the citric acid TCA cycle and respiratory electron transport, pyruvate metabolism and citric acid TCA cycle and mitochondrial TCA cycle enzyme complex sets. **g**. Representative immunofluorescence image for IL-1β staining, nuclear staining (DAPI) and merge in senescent macrophages (DSJ and RSJ) after different concentrations of C-IN-1 stimulation. **h.** Mean fluorescence intensity of IL-1β staining in R, J, DSR, DSJ, RSR, or RSJ, respectively. **i**. Representative immunoblots showing the levels of IL-1β in control and senescent macrophages (DSR, RSR, DSJ and RSJ) after 20 μM C-IN-1 stimulation (left). Quantitative analysis of IL-1β immunoblots in the protein level (right). **j**. ELISA of IL-1β levels in DSJ or RSJ treated with 20 μM C-IN-1. **k**, IL-1β expression analysis under different glutamine-deprivation conditions in DSJ or RSJ. Scale bar, 100 µm (**g**). *n* = 3 in each group from independent biological replicates **(h-k)**. Statistical significance was determined by one-way ANOVA with Tukey’s post-hoc test; mean ± s.d. **(h-k)**.


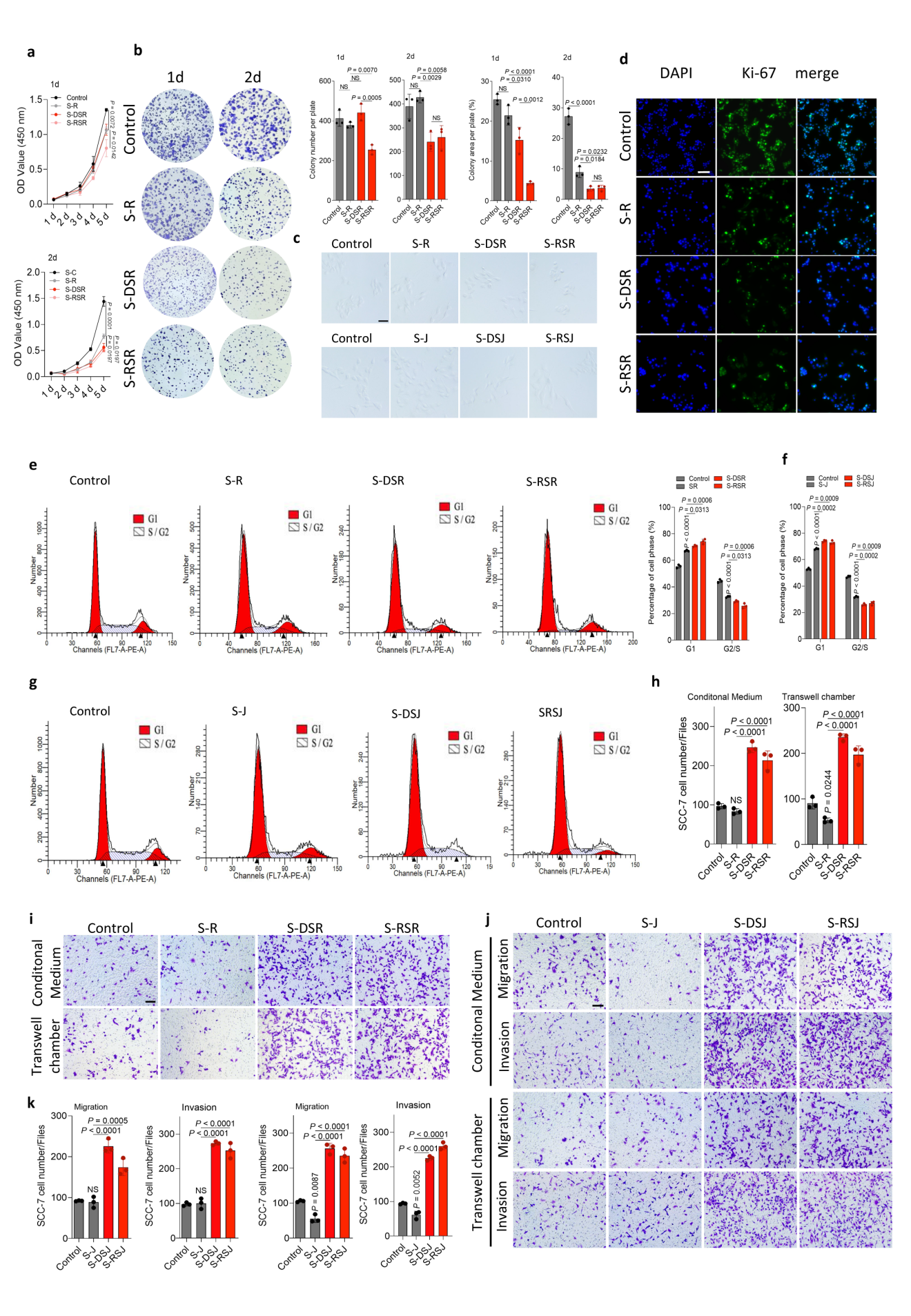


**Fig. S6 | Additional senescent macrophages promote oral cancer invasion *in vitro*.** **a.** OD values at 450 nm of SCC-7 cell culture medium measured after 1 or 2 days of co-culture with blank control, R, DSR or RSR following the addition of the CCK-8 reagent. **b**. Representative colony formation images and quantitation of SCC-7 cells co-cultured with senescent macrophages for 1 or 2 days. Compared with controls, senescent macrophages did not significantly affect colony numbers but reduced colony size. **c.** Representative SA-β-gal staining image for SCC-7 cells after 2 days of co-culture with no significant change. Scale bar, 100 µm. **d.** Representative images showing expression of Ki-67 by immunofluorescence staining in SCC-7 cells after 2 days of DMEM alone, co-culture with macrophages (R, DSR or RSR). Nuclei were stained with DAPI. Scale bar, 100 µm. **e-g.** Flow cytometry showing senescent macrophages inhibit the proliferative ability of SCC-7 cells after 2 days of co-culture, compared to the blank and control groups (R or J). **h, k.** Representative images and quantification of migration in Transwell assays with conditioned medium (CM) or co-culture with R, DSR or RSR, respectively. SCC-7 cells were treated by CM derived from R, DSR or RSR, respectively. Scale bar, 100 µm. **i, j.** Representative images showing migration and invasion levels in Transwell assays with conditioned medium (CM) or co-culture with R, DSR or RSR, respectively. SCC-7 cells were treated by CM derived from J, DSJ, or RSJ respectively. Scale bar = 100 µm. *n* = 3 in each group from independent biological replicates and statistical significance was determined by one-way ANOVA with Tukey’s post-hoc test (**a, b, d, e, f, k**)


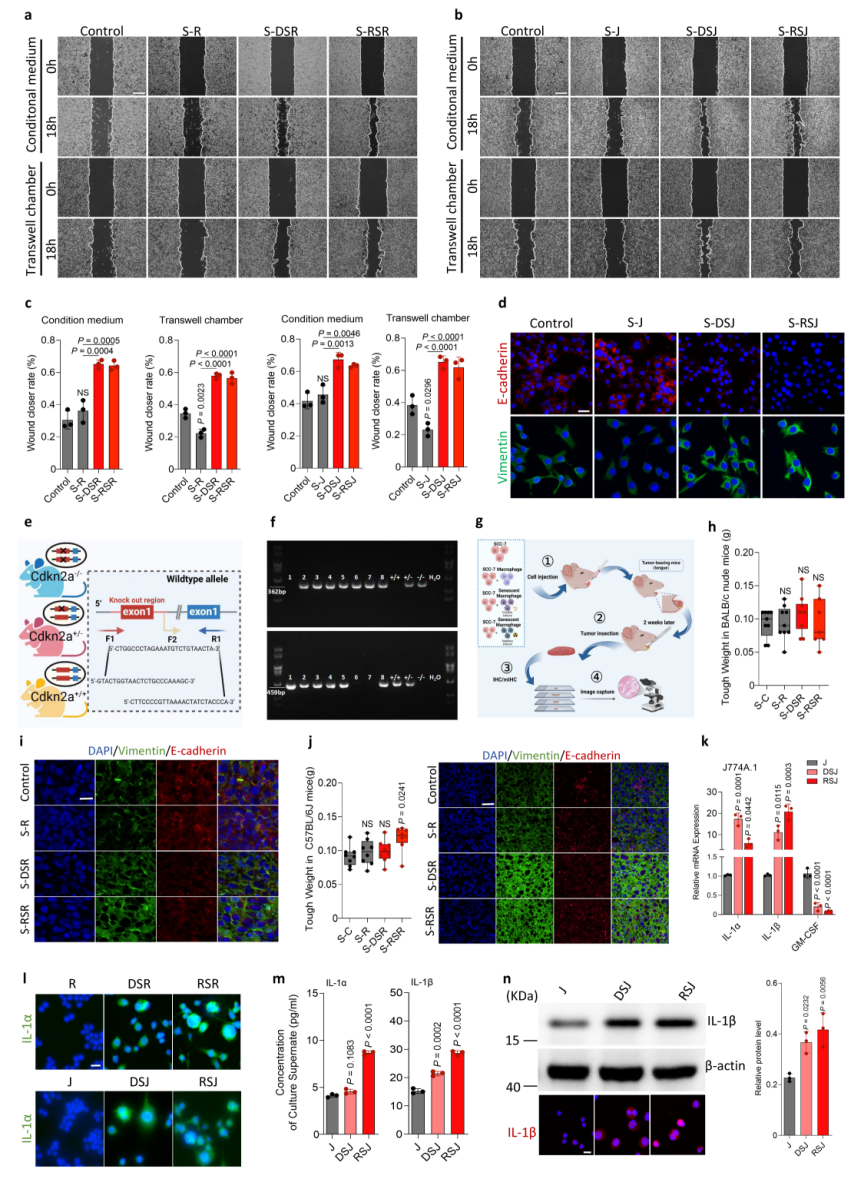


**Fig. S7 | Additional senescent macrophages secrete IL-1β for promoting oral cancer invasion *in vitro* and *in vivo*. a, b.** Representative images showing wound healing levels for SCC-7 cells conditioned medium (CM) or co-culture with senescent macrophages. SCC-7 cells were treated by CM derived from R, DSR or RSR, and J, DSJ, or RSJ respectively. Scale bar, 200 µm. **c.** Wound closure rate (%) within the 18 h for SCC-7 cells co-cultured with a Transwell chamber or CM. **d.** Representative images of immunofluorescence staining of E-cadherin and Vimentin staining in SCC-7 cells co-cultured with a Transwell chamber of in alone, J, DSJ or RSJ, respectively. Scale bar, 50 µm. **e**. Schematic of the *Cdkn2a^-/-^* (*p16^-/-^*) genetic mouse model. To Establish of complete knockout C57BL/6J mice via CRISPR /Cas9 strategies (KO mice), exon1 was removed and identified by PCR. The genotypes were determined to be *Cdkn2a^-/-^* (homozygote), *Cdkn2a^+/-^* (heterozygote) and *Cdkn2a^+/+^* (wild type). **f.** Representative image of *Cdkn2a* knockdown gene mouse identification. The numbers 1 were *Cdkn2a^+/+^* wild type mice, the numbers 2, 3, 4, 5, and 8 were heterozygous *Cdkn2a^-/-^* genetic mice, and the numbers 6, 7 were homozygous *Cdkn2a^-/+^* genetic mice. **g.** Schematic of the experimental approach for establishing a transplanted tumour model in the tongue. **h-i.** Representative immunofluorescent images with co-expression of E-cadherin and Vimentin and tongue weight in BALB/c nude mice bearing tumours from SCC-7 cells alone (*n* = 9) or with R (*n* = 9), DSR (*n* = 10), RSR (*n* = 7), respectively. Scale bar, 20 µm. **j.** Representative immunofluorescent images with co-expression of E-cadherin and Vimentin and tongue weight in *p16*^-/-^ C57BL/6J mice bearing tumours from SCC-7 cells alone (*n* = 8) or with R (*n* = 8), DSR (*n* = 8), RSR (*n* = 8), respectively. Scale bar, 20 µm. **k**. Relative mRNA expression of GM-CSF, IL-1α, and IL-1β in the DSJ or RSJ from J774A.1 cells, respectively. **l**. Representative immunofluorescence staining images of IL-1α levels in the intracellular protein of R, DSR, and RSR, or J, DSJ, and RSJ. Scale bars, 50 µm. **m.** Upregulated IL-1β levels in the supernatants of DSJ and RSJ were determined by ELISA. **n.** Immunoblots and representative immunofluorescence staining images of IL-1β levels in the intracellular protein of J, DSJ, and RSJ. Scale bars, 50 µm. *n* = 3 in each group from independent biological replicates and statistical significance was determined by one-way ANOVA with Tukey’s post-hoc test; mean ± s.d. (**c, m, n**).


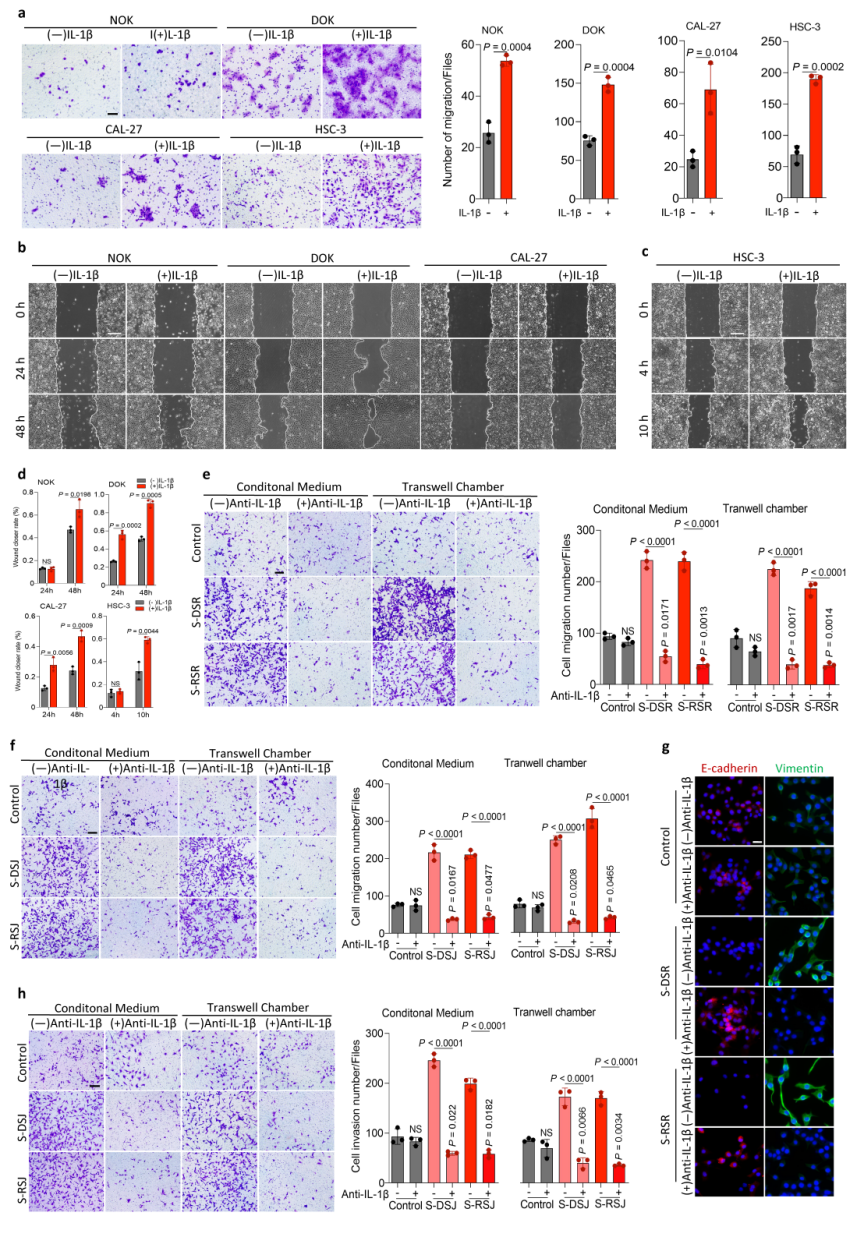


**Fig. S8 | Additional exogenous recombinant IL-1β promotes oral cancer invasion and blocking IL-1β attenuates this process and downstream signaling pathway. a.** Representative images and quantification of migration assays. Human derived cell lines including normal oral keratinocytes (NOK), dysplastic oral keratinocytes (DOK), tongue squamous cell carcinoma cells (CAL-27) and OSCC cells (HSC-3) were treated with exogenous IL-1β. Scale bar, 100 µm. **b-d.** Representative images and quantification showing wound healing levels in NOK, DOK, CAL-27 and HSC-3 treated by recombinant IL-1β. Scale bar, 100 µm. **e**. Representative images and quantification of migration assays after treatment with DSR or RSR with an anti-IL-1β antibody. Scale bar, 100 µm. **f, h**. Representative images and quantification of migration (**f**) and invasion (**h**) assays after treatment with DSJ or RSJ with an anti-IL-1β antibody. Scale bar, 100 µm. **g**. Representative images of immunofluorescence staining of E-cadherin and Vimentin in SCC-7 co-cultured indirectly with DSJ or RSJ treated with or without anti-IL-1β antibody, respectively. Scale bars, 100 µm. *n* = 3 in each group from independent biological replicates and statistical significance was determined by one-way ANOVA with Tukey’s post-hoc test; mean ± s.d. (**a, d, e, f, h**).

**
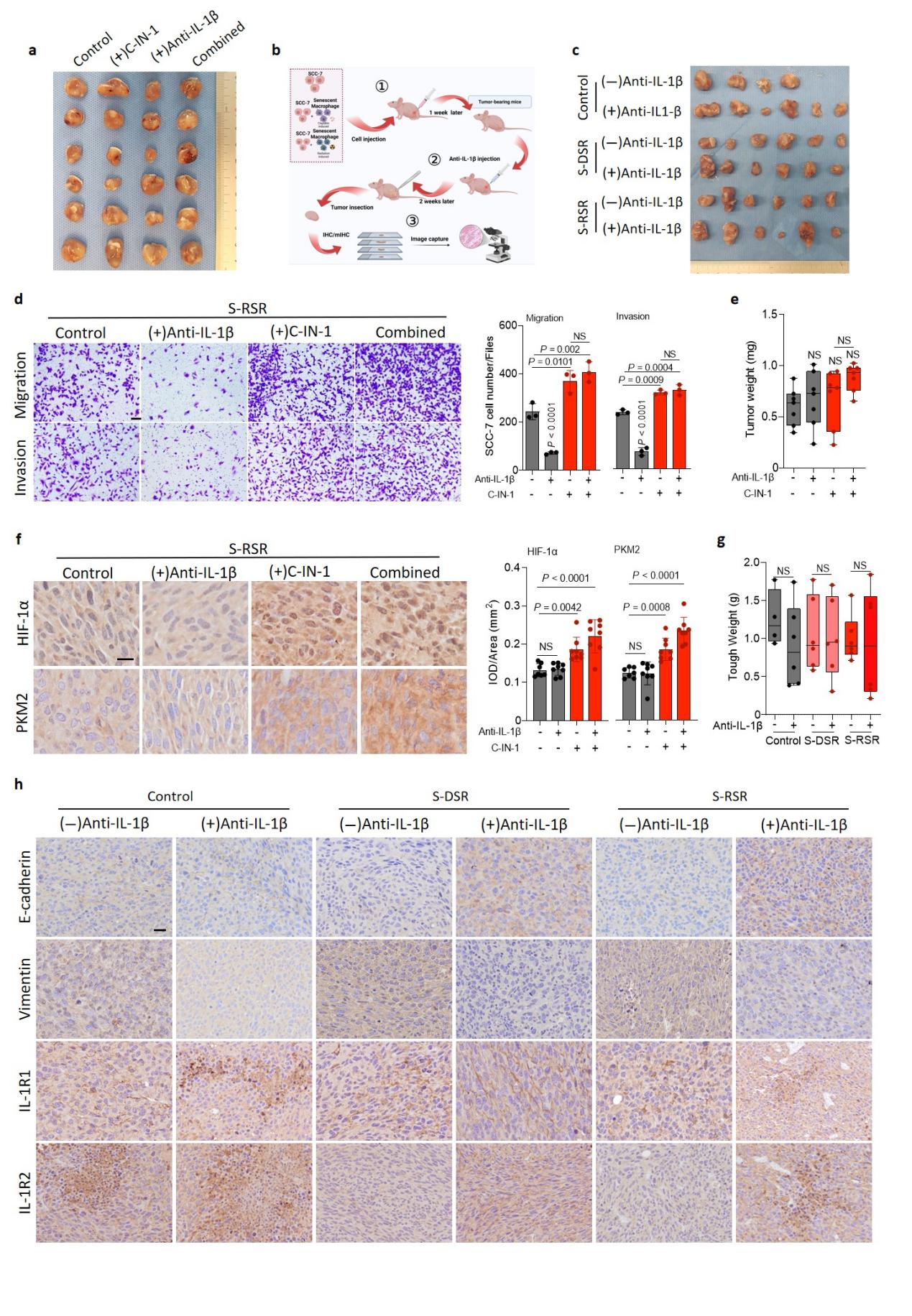
**

**Fig S9 | Additional inhibition of IL-1β reduces vimentin and increases E-cadherin to suppress OSCC invasion *in vivo*. a.** Representative images of tumour sections from S-RSR treated by anti-IL-1β antibody alone, C-IN-1 alone, and a combination with anti-IL-1β antibody and C-IN-1. **b.** Schematic of the experimental approach for establishing a xenograft mouse model. First, SCC-7 cells were injected subcutaneously with DSR or RSR for tumour formation. Secondly, an anti-IL-1β antibody suspended in 15% w/v PLGA-PEG-PLGA gel was injected around the tumour site one week later. Two weeks later, the tumour samples were harvested for IHC and mIHC staining procedures. **c.** Representative images of tumours from nude mice implanted with SCC-7 cells alone and co-implanted with DSR or RSR treated with vehicle or anti-IL-1β antibody. **d.** Representative images and quantification showing Transwell migration and invasion levels for SCC-7 cells co-cultured RSR in a Transwell chamber treated by anti-IL-1β antibody alone, C-IN-1 alone, and a combination with anti-IL-1β antibody and C-IN-1. **e.** Tumour weight from nude mice co-implanted with SCC-7 cells and RSR. **f.** Representative images and quantification of HIF-1α and PKM2 in tumour sections from S-RSR treated by anti-IL-1β antibody alone, C-IN-1 alone, a combination with anti-IL1-β antibody and C-IN-1 (*n* = 7 independent biological replicates for each group). **g.** Tumour weight of nude mice from SCC-7 cells alone and co-implanted with DSR or RSR treated with vehicle or anti-IL-1β antibody (*n* = 6 in the comparisons except *n* = 4 in the group of SCC-7 cells alone + vehicle). **h.** Representative IHC images of E-cadherin and vimentin, or IL-1R1 and IL-1R2 in nude mice bearing tumour from SCC-7 cells alone, S-DSR or S-RSR treated with or without the anti-IL-1β antibody. Statistical significance was determined by one-way ANOVA with Tukey’s post-hoc test; mean ± s.d. **(d, e, f, g**).
